# Machine Learning Methods for Diagnosing Autism Spectrum Disorder and Attention- Deficit/Hyperactivity Disorder Using Functional and Structural MRI: A Survey

**DOI:** 10.3389/fninf.2020.575999

**Published:** 2021-01-20

**Authors:** Taban Eslami, Fahad Almuqhim, Joseph S. Raiker, Fahad Saeed

**Affiliations:** ^1^Department of Computer Science, Western Michigan University, Kalamazoo, MI, United States; ^2^School of Computing and Information Sciences, Florida International University, Miami, FL, United States; ^3^Department of Psychology, Florida International University, Miami, FL, United States

**Keywords:** machine learning, deep learning, Attention Deficit and Hyperactivity Disorder (ADHD) classification, Autism Spectrum Disorder (ASD) classification, diagnosis, sMRI, fMRI, survey

## Abstract

Here we summarize recent progress in machine learning model for diagnosis of Autism Spectrum Disorder (ASD) and Attention-deficit/Hyperactivity Disorder (ADHD). We outline and describe the machine-learning, especially deep-learning, techniques that are suitable for addressing research questions in this domain, pitfalls of the available methods, as well as future directions for the field. We envision a future where the diagnosis of ASD, ADHD, and other mental disorders is accomplished, and quantified using imaging techniques, such as MRI, and machine-learning models.

## 1. Introduction

Modern techniques to diagnose mental disorders were first established in the late 19th century (Laffey, 2003) but its genesis can be traced back to 4th century BCE (Elkes and Thorpe, 1967). Gold standard for diagnosing most mental-disorders rely primarily on information collected from various informants (e.g., parents, teachers) regarding the onset, course, and duration of various behavioral descriptors that are then considered by providers when conferring a diagnostic decision based on DSM-5/International Classification of Diseases-10th Edition (ICD-10) criteria (World Health Organization, 2004; Pelham et al., 2005; American Psychiatric Association, 2013). The methods used by providers to obtain this information range from relatively subjective (e.g., rating scales) and unstructured (e.g., unstructured clinical interviews) to more objective (e.g., direct observations) and structured (e.g., structured diagnostic interviews) approaches.

Autism Spectrum Disorder (ASD) and Attention-Deficit/Hyperactivity Disorder (ADHD) are prevalent brain disorders among children which usually persist into adulthood. ASD is a neurodevelopmental disorder characterized by communication, behavior and social interaction deficits in patients which may include repetitive behavior, irritability, and attention problems (Maenner et al., 2020). Since the introduction of the 5th edition of the Diagnostic and Statistical Manual of Mental Disorders (DSM-5), ASD has reflected a larger umbrella diagnostic entity that was previously reflective of multiple discrete disorders including Autistic Disorder, Asperger's syndrome, and other Pervasive Developmental Disorders (Kogan et al., 2009). Recent studies suggest that the prevalence of ASD among children has increased from 1 in 100 to 1 in 59 over 14 years (from the year 2000 to 2014) (Maenner et al., 2020). ADHD is also a common brain disorder among children which causes problems, such as hyperactivity, impulsivity, and inattention. Like ASD, ADHD often continues to adulthood (Sibley et al., 2017). Approximately 5–9.4% of children are diagnosed with the disorder (Polanczyk et al., 2007; Danielson et al., 2018). Prevalence of ASD, and ADHD in children necessitates accurate and timely identification, and diagnosis of these disorders.

Current practice guidelines for the assessment, diagnosis, and treatment of ADHD, and ASD recommend an approach that adheres to the Diagnostic and Statistical Manual (DSM) symptom criteria (Wolraich et al., 2019) with an emphasis on verifying that symptoms occur across more than one setting (e.g., home, school). These practice guidelines highlight the importance of ruling out the presence of co-occurring and/or alternative diagnoses (e.g., anxiety, mood disorders, learning problems) that share notable features with ADHD (e.g., difficulty concentrating) or ASD further complicating diagnostic assessment. Despite the development and revision of these practice guidelines for over two decades (Perrin et al., 2001); there is evidence of substantial variability in the extent to which these practice guidelines are implemented in routine clinical care in the diagnosis of the disorder (Epstein et al., 2014). Lack of uniformity in adoption of these practice guidelines has the potential to result in over-, under-, and/or misdiagnosis of the disorder (for a review, see Sciutto and Eisenberg, 2007). In fact, Bruchmüller et al. (2012) demonstrated that a sizeable number of professionals fail to adhere to DSM or International Classification of Diseases (ICD) criteria altogether when diagnosing the disorder. Specifically, an average of 16.7% providers participating in their study assigned a diagnosis of ADHD to an example patient despite multiple criteria missing and/or the child presenting with a different diagnosis altogether. Follow-up analyses among only those providing a diagnosis (rather than deferring their diagnostic decision due to lack of information) revealed a false positive rate of nearly 20%. While exact estimates of misdiagnosis of ADHD, and ASD are not available, if the results of this study reflect typical clinical practice and nearly 1/5 of children diagnosed with ADHD or ASD in the population are currently misdiagnosed (impacting one million children in the US). These children may fail to obtain treatment for other diagnoses they have (e.g., anxiety disorders) or receive treatments that are unnecessary (Danielson et al., 2018), resulting in financial burden (Pelham et al., 2007), and may result in snatching-away services actually needed by children with the disorders (Raiker et al., 2017). Other obstacles for diagnosis include disagreement between parent- and teacher-rated perceptions (Narad et al., 2015), substantial time-commitment required for interviews (Pelham et al., 2005), and malingering/faking symptoms of ADHD, and ASD especially in adulthood (Musso and Gouvier, 2014). Collectively, these limitations have resulted in calls for more optimal assessment methods for psychological disorders, such as cognitive (e.g., tasks) or neurobiological (e.g., imaging) methods (Linden, 2012; Castellanos et al., 2013).

In the 1990's, advent of magnetic resonance imaging (MRI) allowed one to directly study the brain activity without requiring people to undergo injections, surgery, ingest substances, or to be exposed to ionizing radiation. It was also considered potentially more objective than other quantitative methods, such as continuous performance tests (Inoue et al., 1998; Nichols and Waschbusch, 2004; Faraone et al., 2016; Park et al., 2019) or rating scales (Bruchmüller et al., 2012; Raiker et al., 2017). Suddenly, computational scientists with little or no training in psychiatry or psychology could analyze data collected from imaging methods and make inferences for patients with mental disorders (Castellanos and Aoki, 2016).

**Figure 1:**
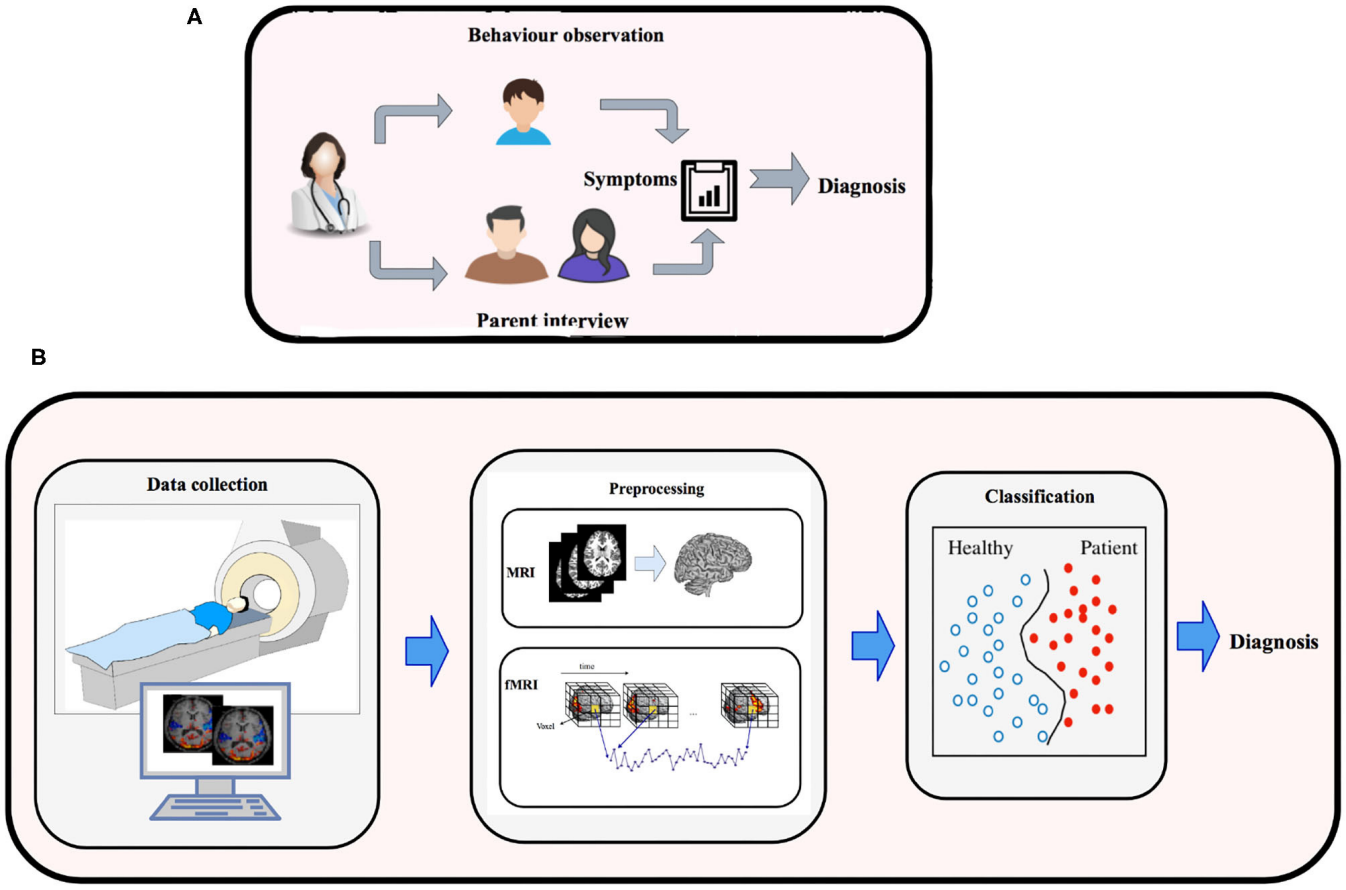
**(A)** Traditional methods for diagnosing brain disorders vs. **(B)** classification based on brain imaging and machine learning.

Machine-learning is a subfield of Artificial Intelligence, that has the potential to substantially enhance the role of computational methods in neuroscience. This is apparent by substantial work that has been carried out in developing machine-learning models, and deep-learning techniques to process high-dimensional MRI data to model neural pathways that govern the brains of various mental disorders (Vieira et al., 2017). These efforts have resulted in development of machine-learning methods to classify Alzheimer's, Mild Cognitive impairment (Duchesnay et al., 2011), Temporal Lobe Epilepsy, Schizophrenia, Parkinson (Bind et al., 2015), Dementia (Ye et al., 2011; Ahmed et al., 2018; Pellegrini et al., 2018), ADHD (Eslami and Saeed, 2018b; Itani et al., 2019), ASD (Pagnozzi et al., 2018; Eslami et al., 2019; Hyde et al., 2019), and major depression (Gao et al., 2018). These machine-learning models rely on statistical algorithms, and are suitable for complex problems involving combinatorial explosion of possibilities or non-linear processes where traditional computational models fail in quality or scalability. Figure 1 shows the traditional approach (outlined above) vs. quantitative ML methods (outlined below) for diagnosing brain disorders.

### 1.1. Motivation for Machine-Learning to Guide the Diagnostic Processes, Related Work, and Contributions of This Paper

As discussed above, the presence of certain behavioral characteristics, such as attention problems do not always indicate a specific diagnostic entity (e.g., ADHD) given that nebulous symptoms, such as attention problems occur across a variety of disorders (e.g., depression, ASD, anxiety). As a result, conferral of a diagnosis based on DSM-5 or ICD-10 criterion ascribes an underlying cause to the various behavior or emotional difficulties without a method available to verify that the disorder arises from underlying biological dysfunction. Collectively, the absence of specific physiological, cognitive, or biological *validation* creates a host of challenges regarding our ability to confirm existing diagnostic approaches (Saeed, 2018).

Recent advances in neuroscience and brain imaging have paved the way for understanding the function and structure of the brain in more detail. Traditional statistical methods for analyzing brain images relied on mass-univariate approaches. However, these methods overlook the dependency among various regions which now are known to be a great source of information for detection of different brain disorder (Vieira et al., 2017). ML models, on the other hand, are usually working with the relationship among various brain regions as their feature vectors and hence are preferred over other methods. Notably, given the relative infancy of the integration of neuroscience into clinical psychology to better understand disorders, such as ASD and ADHD, the specific brain regions associated with these clinical disorders and their patterns of interaction are not well-known. It is likely that the application of ML methods to neuroimaging data in these populations will result in improved understanding of patterns of neurobiological functioning that would not otherwise be detectable using other methods. These advances will ultimately improve not only our ability to diagnose these disorders but also augment our understanding of the mechanisms that contribute to their etiology.

In this survey, we provide a comprehensive report on ML methods used for diagnosis of ASD and ADHD in recent years using MRI data sets. To the best of our knowledge, there is no comprehensive review covering the recent machine learning methods for ASD and ADHD disorders based on both fMRI and sMRI data. Besides (f)MRI, other types of brain data generated using technologies, such as electroencephalogram (EEG) and Positron Emission Tomography (PET) are used for studying ADHD and ASD (Duchesnay et al., 2011; Tenev et al., 2014; Bosl et al., 2018). It is worth mentioning that based on the effects of ASD on the social interactions of subjects, facial expression, and eye-tracking measurements have been used to evaluate the utility of machine learning models in accurately classifying individuals with and without ASD (Liu et al., 2016; Jaiswal et al., 2017). Similarly, given the well-documented neurocognitive dysfunction and alterations in temperament characteristic of individuals with ADHD, graph theoretical and community detection algorithms have been applied to advance our understanding of these deficits in ADHD (Fair et al., 2012; Karalunas et al., 2014). Personal Characteristic Data (PCD), and its integration with MRI data has also shown to give superior performances (Brown et al., 2012; Ghiassian et al., 2016; Parikh et al., 2019) for classification for ADHD and ASD data sets. However, only MRI based machine-learning techniques (for ADHD and ASD) will be considered within the limited scope of this review.

In this paper, we organize, and present the applications of machine-learning for MRI data analysis used for identification, and classification of ADHD and ASD. The paper will give a broad overview of the existing techniques for ASD and ADHD classification, and will allow neuroscientists to walk through the methodology for the design and execution of these models. We start by reviewing the basics of machine-learning, and deep-learning strategies. Wherever possible we will use MRI data as an example when explaining these concepts. In next sections, we will identify the motivation and areas where (and why) these machine-learning models can make an impact in mental diagnosis. Lastly, we discuss in some detail the progress that has been made in developing machine-learning solutions for Autism Spectrum Disorder (ASD) and Attention-Deficit/Hyperactivity Disorder (ADHD), Identifying challenges and limitations of current methods, and suggestions and directions for future research.

## 2. Introduction to Machine Learning and Deep Learning

Machine-Learning (ML) is a subset of artificial intelligence that gives the machine the ability to learn from data without providing specific instructions (Alpaydin, 2016). Machine Learning is divided into three broad categories: supervised learning, unsupervised learning, and semi-supervised learning. The goal of supervised learning (Caruana and Niculescu-Mizil, 2006) is to approximate a function *f* which maps the input *x* to output *y* in which *x* refers to training data and *y* refers to labels which could be discrete/categorical values (classification) or continues values (regression). Unlike supervised learning, in unsupervised learning (Hinton et al., 1999), there is no corresponding output for the input data. The goal of unsupervised learning is to draw inference and *learn* the structure and patterns of the data (Radford et al., 2015). Cluster analysis is the most common example of unsupervised learning. Semi-supervised learning (Zhu and Goldberg, 2009) is a category of ML which falls between supervised and unsupervised learning. In semi-supervised learning techniques, unlabeled data is used for learning the model along with labeled data (Chapelle et al., 2009).

**Figure 2:**
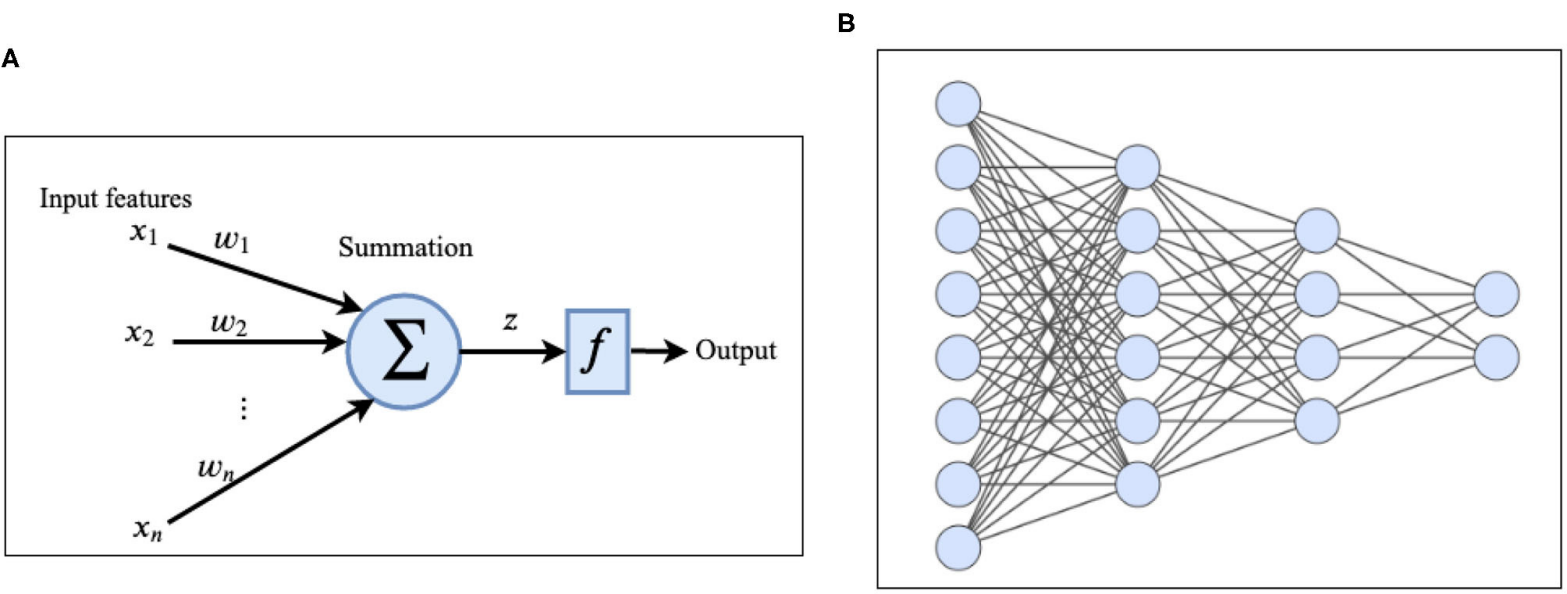
**(A)** Architecture of an artificial neuron. **(B)** Example of a deep feed forward network with two hidden layers.

Deep-Learning (DL) (Goodfellow et al., 2016) is a branch of ML which is inspired by the information processing in the human brain. A deep neural network (DNN) (LeCun et al., 2015) consists of one input layer, several hidden layers, and one output layer. Hidden layers are responsible for extracting useful features from the input data. Each layer consists of several units/nodes called artificial neurons (Krizhevsky et al., 2012) (Figures 2A,B). The simplest type of deep neural network is a *deep feed forward network* in which the nodes in each layer are connected to the nodes in the next layer (Glorot and Bengio, 2010). There is no cycle and no connection between nodes in the same layer and as the name implies, information flows forward from the input layer to the output layer of the network. Multi-layer-perceptron (MLP) (Hornik et al., 1989; Gardner and Dorling, 1998) is a specific type of feed-forward network in which each node is connected to all the nodes in the next layer. Each node receives the input from nodes in the previous layer, applies some linear and non-linear transformations and transmits it to the next layer. The information is propagated (Rumelhart et al., 1986) through the network over the weighted links that connect nodes of consecutive layers. Activation of the node *z* at each layer can be computed using the following equation:

(1) $$\begin{array}{l} a=\sigma \left(\overset{m}{\underset{i=1}{\sum }}w_{i}x_{i}+b\right) \end{array}$$

In which *x* corresponds to values of nodes in the previous layer, *w* corresponds to the weights of connections between node *z* and nodes in the previous layer, *b* corresponds to bias and σ is a non-linear activation function. Non-linear activation functions (Huang and Babri, 1998) are essential parts of neural networks that enable them to learn non-linear and complicated functions. Sigmoid, tangent hyperbolic (tanh) (Schmidhuber, 2015), and rectified linear (ReLU) (Nair and Hinton, 2010) are the most used activation functions in neural networks. Vargas et al. (2017) state that number of deep learning publications increased from 39 to 879 between 2006 and 2017. Similarly, the application of deep-learning models applied for identification and diagnosis of mental-disorders have increased rapidly in recent years. In the following section we focus on description of deep-learning models, methods, and techniques to make it more accessible to neuroscientists.

### 2.1. Training of a Deep-Learning Model

The set of weights and biases of the network are known as its parameters or degrees of freedom which should be optimized during the training process. Training a neural network starts by assigning random parameters to the network. The input data is propagated to the network by applying a non-linear transformation using Equation (1). The input of each intermediate layer of the network is the output of its previous layer. Finally, the prediction error is calculated in the output layer by applying a loss function to the predicted value and ground truth. Depending on the type of problem and the output, appropriate loss functions should be considered. For example, Mean Squared Error (MSE) and Mean Absolute Error (MAE) are well-known functions in regression problems (Prasad and Rao, 1990). Another example is cross entropy loss which is used for multi-class classification. The error computed using the loss function is used to update the parameters of the model in order to reduce the prediction error. The most famous algorithm for training the neural networks is called backpropagation (Hecht-Nielsen, 1989; Rezende et al., 2014). Backpropagation is based upon an optimization algorithm called stochastic gradient descent (SGD) (Bottou, 2012) which changes the values of the network parameters by computing the gradient of the loss function with respect to each of them using the chain rule. The value of each parameter is increased or decreased in order to reduce the prediction error of the network. This process is repeated several times during the training process until training loss becomes below a threshold or a maximum number of iterations is reached. After the training process, the network is ready to use for predicting the output of unseen data (test set).

### 2.2. Overfitting in Neural Networks

Over-fitting is one of the major issues in DL that causes the model to fit very well to the training data but performs poorly for unseen data. Deep neural networks usually contain many parameters, millions in the case of very deep networks (Krizhevsky et al., 2012; Szegedy et al., 2015) which causes the over-fitting problem. This is particularly problematic with respect to generalizing findings to a clinical setting. Specifically, given that the actual diagnostic status of patients (i.e., they have/do not have the disorder) is unknown at the time of presentation it is critical that the adoption of DL methods and the integration of neuroimaging are applicable to new cases rather than cases included in research samples. Fortunately, over-fitting can be prevented by using regularization methods. Regularization is a class of approaches the reduce the generalization error of a network without reducing its training error by adding some modifications to the learning process. Some of the most well-known regularization methods are as follows:

#### 2.2.1. L1/L2 Regularization

L1 and L2 regularization are one of the most popular regularization methods in which a regularization term is added to the cost function. Equations (2) and (3) show the L1 and L2 regularization terms:

(2) $$\begin{array}{l} \frac{\lambda }{2}\|w\|=\frac{\lambda }{2}\overset{m}{\underset{j=1}{\sum }}w_{j} \end{array}$$

(3) $$\begin{array}{l} \frac{\lambda }{2}{\left\|w\right\|}^{2}=\frac{\lambda }{2}\overset{m}{\underset{j=1}{\sum }}w_{j}^{2} \end{array}$$

In this equations λ is the regularization parameter. Adding these equations to the cost function penalized the value of network weights and therefore leads it to a simpler model and avoids the overfitting.

#### 2.2.2. Drop-Out

Dropouts ignore some of the units (and their corresponding connections) randomly in the training process which as a result reduces the number of parameters of the model (Srivastava et al., 2014).

#### 2.2.3. Batch Normalization

Batch normalization stabilizes the training of deep neural networks, which helps faster convergence. Initially, BN was proposed to reduce the internal covariance shift, but later, Santurkar et al. (2018) studied the effect of BN and concluded that the effect of BN is mainly on smoothening the landscape. In this method, the output of each activation layer is normalized by subtracting the mean and dividing it to the standard deviation of the batch. Batch normalization regularizes the model and hence can reduce its overfitting (Ioffe and Szegedy, 2015).

## 3. Magnetic Resonance Imaging (MRI), and Feature Extraction

Functional magnetic resonance imaging or functional MRI (fMRI) is a non-invasive technique that measures the brain activity by detecting changes associated with blood flow (Logothetis et al., 2001). The techniques exploits the fact that cerebral blood flow and neural activity are correlated, i.e., blood flow in the brain where neurons are firing.

Structural MRI (sMRI) is also a non-invasive technique that provides sequences of brain tissue contrast by varying the excitation, and the repetition times to image different structure of the brain. These sequences produce volumetric measurements of the brain structure (Bauer et al., 2013). Similar to fMRI, sMRI data has shown to contain quantifiable biomarkers and features, such as early circumference enlargement and volume overgrowth of the brain, that can be used as the input to machine learning models for detection of brain disorders.

### 3.1. Defining Features for Classification Using Functional MRI (fMRI) Data

An important step for designing a solution using ML models is deriving features from the data. Although substantial work dedicated to understanding the neurobiological underpinnings of both ADHD and ASD is ongoing, pinpointing the exact neurobiological correlates remains a challenge creating difficulties related to optimal feature selection. Fortunately, several approaches outlined below have been developed to assist in this endeavor. fMRI based features are extracted from the time series of voxels or regions of interest (ROI). ROIs can be defined based on structural properties like anatomical atlases or functional features of fMRI time series using clustering algorithms. These methods can also be applied to the components generated using Independent Component Analysis (ICA) method. ICA is a data analysis method that finds the maximally independent components of the brain without explicit prior knowledge (Calhoun et al., 2001). In the following sub-section, we explain the most frequently used methods for defining ML features.

#### 3.1.1. Functional Connectivity

One of the concepts that is widely used to generate features from fMRI data is the strength of functional connections between pairs of regions. Functional connectivity between two regions of the brain can be approximated using different measures as explained below:

Pearson's correlation

Pearson's correlation is the most used measure for approximating functional connectivity. It works well to measure the linear association between two time series, *v* and *u*, and mostly is calculated using the following equation: The Pearson's correlation between variables *v* and *u* is calculated using the following equation:

(4) $$\begin{array}{l} \rho_{uv}=\frac{\sum_{t=1}^{T}\left(u_{t}-\bar{u}\right)\left(v_{t}-\bar{v}\right)}{\sqrt{\sum_{t=1}^{T}{\left(u_{t}-\bar{u}\right)}^{2}}\sqrt{\sum_{t=1}^{T}{\left(v_{t}-\bar{v}\right)}^{2}}} \end{array}$$

Spearman's rank correlation

Unlike Pearson's correlation, Spearman's rank correlation measures the strength of a monotonic association between two variables. Spearman's rank correlation works well when the variables are rank-ordered. This measure calculates the Pearson's correlation between the ranked values of variables u and v. In the case of distinct ranks in the data, Spearman's correlation can be calculated using the following equation: This measure calculates the Pearson's correlation between the ranked values of variables *u* and *v*. In the case of distinct ranks in the data, Spearman's correlation can be calculated using the following equation:

(5) $$\begin{array}{l} \rho_{uv}=1-\frac{6\sum d_{i}^{2}}{n\left(n^{2}-1\right)} \end{array}$$

In this equation *d* corresponds to the difference between two corresponding ranks.

Mutual information

Another measure for estimating the functional connectivity is the mutual information between two-time courses which can be computed using the following equation:

(6) $$\begin{array}{l} MI\left(u,v\right)=\underset{u\in S_{u}}{\sum }\underset{v\in S_{v}}{\sum }p\left(u,v\right)\log\left(\frac{p\left(u,v\right)}{p\left(u\right)p\left(v\right)}\right) \end{array}$$

#### 3.1.2. Dynamic Functional Connectivity

**Figure 3:**
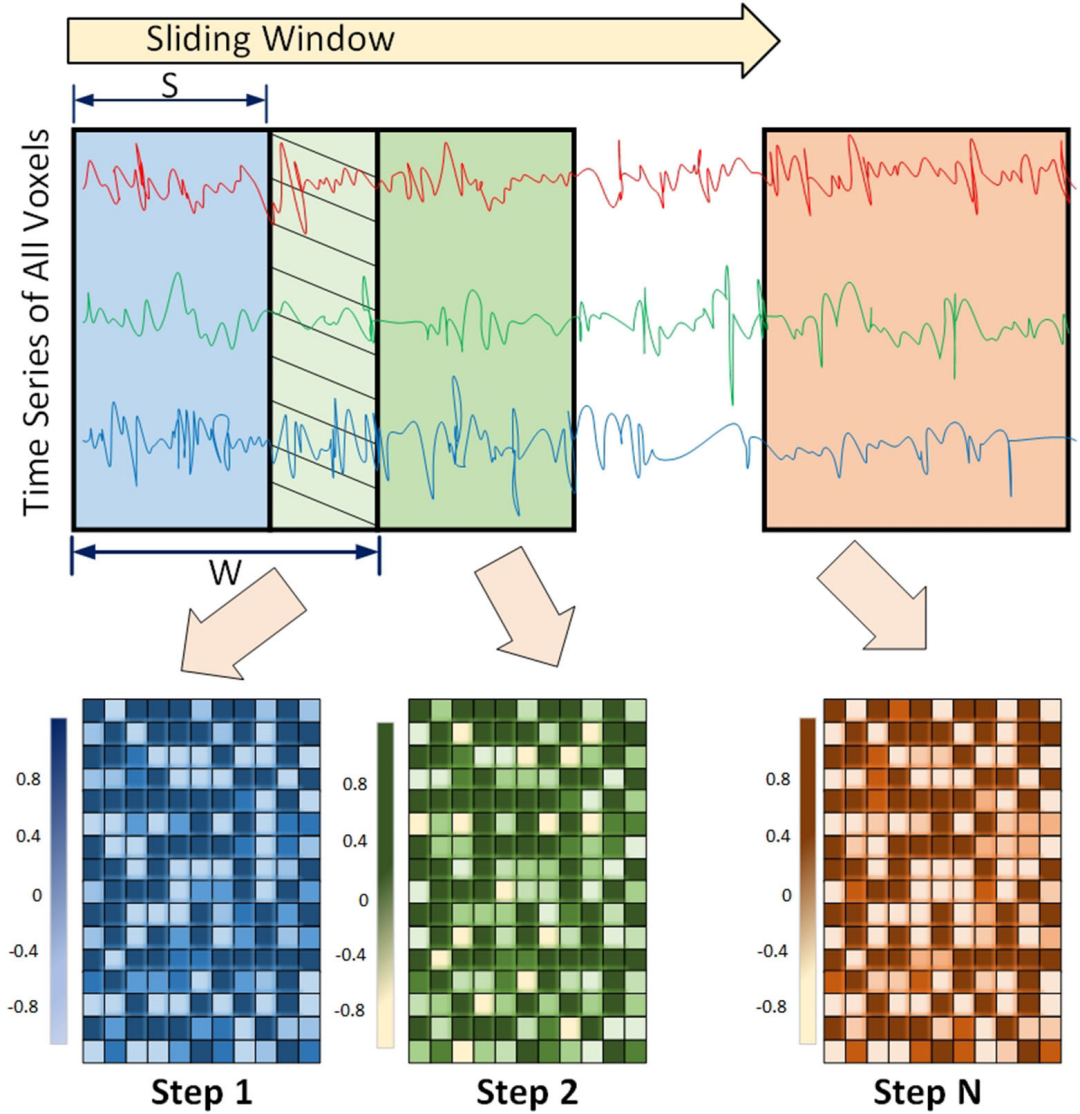
Sliding window Framework for computing Dynamic functional connectivity (DFC) is shown. DFC is an expansion of traditional functional connectivity and assumes that functional connectivity changes over a short time - leading to more richer analysis of fMRI data using machine-learning models.

Functional connectivity among regions of the brain is shown to have a dynamic behavior rather than being static. This means that the strength of association between the two regions may change over time. This concept is called Dynamic Functional Connectivity (DFC), and is shown to be increasingly important in understanding cognitive processing (Chen et al., 2020b; Kinany et al., 2020; Liu et al., 2020; Premi et al., 2020), and mental disorders including ADHD (Kaboodvand et al., 2020), and ASD (Mash et al., 2019; Rabany et al., 2019). This dynamic behavior is usually detected using a sliding window framework. In this framework, a window of size *w* slides over the time series and functional connectivity among all regions are computed based on the covered time points in the window. The window slides over *s* elements and covers the next consecutive *w* time points. This process is repeated until the window reaches the end of the time series (Preti et al., 2017). An example of the sliding window framework is shown in Figure 3.

#### 3.1.3. Graph Theoretical Measures

The array consisting of all pairwise correlations is usually considered as the feature vector for training ML models. Alternatively, the correlation among various regions can be used to construct a graph called the brain *functional network*. After removing weak correlations based on a predefined threshold, remaining correlations define the edges connecting brain regions to each other. This graph can be considered as a weighted graph (strength of correlations as edge weights) or an unweighted graph. Computing the properties of brain functional network, such as degree distribution (Iturria-Medina et al., 2008), clustering coefficient (Supekar et al., 2008), closeness centrality (Lee and Xue, 2017), etc., represents another method for defining features from fMRI data which has been widely used in brain disorder diagnosis (Colby et al., 2012; Brier et al., 2014; Khazaee et al., 2015; Openneer et al., 2020). Examples of graph-theoretical properties used in the literature are provided in Supplementary Table 1.

#### 3.1.4. Frequency Properties

Another practice for extracting features from fMRI data is applying Fast Fourier Transformation (FFT) to time series of each voxel/region and transform the data from the time domain to frequency domain (Kuang and He, 2014; Kuang et al., 2014). For each voxel/region, the frequencies associated with the highest value of amplitudes are selected as the feature from fMRI data.

### 3.2. Defining Features for Classification Using Structural MRI (sMRI) Data

In this section, we broadly discuss the most commonly used methods for defining features from sMRI data.

#### 3.2.1. Morphometric Features

High resolution images generated using the MRI technology provide detailed information about the structure of the brain. Different morphometric attributes, such as *volume, area, thickness, curvature*, and *folding index* of different regions are widely used as the features of each subject for the classification task. These features can be easily extracted from tools, such as FreeSurfer. FreeSurfer is an open-source tool that is automated to extract key features in the brain by providing a full preprocessing to obtain morphometric features. The preprocessing includes skill stripping, gray-white matter segmentation, reconstruction of the cortical surface, and region labeling (Fischl, 2012).

#### 3.2.2. Morphological Networks

Interconnectivity between morphological information of brain regions is another way for defining feature vectors. In Wang et al. (2018), morphological connectivity is defined as $1-\frac{d\left(x_{i},x_{j}\right)}{D}$ in which *x*_*i*_ refer to a vector containing morphometric features of regions *i*, such as cortical thickness, cortical curvature, folding index, brain volume, and surface area, *d* refers to mahalanobis distance and *D* is an integer value. Similarly, Kong et al. (2019) compute the connectivity between two ROIs based on their gray matter volume using the equation $\frac{1}{d\left(a,b\right)+1}$ in which *d*(*a, b*) = |*t*(*a*) − *t*(*b*)|^2^ where *t*(*a*) corresponds to the gray matter volume of region *a*. Similarity-Based Extraction of Graph Networks using Gray Matter MRI Scans are also shown (Tijms et al., 2012; Seidlitz et al., 2018) to provide robust, and biologically plausible individual structural connectomes (Khundrakpam et al., 2019) from human neuroimaging.

## 4. Detection of ASD/ADHD Using Conventional ML Methods

The identification of relevant features utilizing the methods described above allow for further examination of the extent to which these features may aid in the diagnosis of ADHD and ASD. In this section, we provide an overview of studies that used the conventional machine learning methods, such as SVM and Random forest for classification of ASD and ADHD.

### 4.1. ADHD Classification

SVM has been evaluated extensively in the classification of ADHD using fMRI and MRI data. In dos Santos Siqueira et al. (2014) a functional brain graph is constructed by computing Pearson's correlation between time series of each pair of regions, centrality measures of the graph (degree, closeness, betweenness, Eigenvector, and Burt's constraint) are considered as features, and SVM is used for the classification and the highest achieved accuracy across multiple sites was 65%, while a site-by-site accuracy was 77%. In Chang et al. (2012), features from structural MRI are extracted using isotropic local binary patterns and are fed to SVM classifier. The isotropic local binary pattern (LBT) is a powerful technique used in computer vision. LBT is computed in three steps; picked a pixel with its neighborhood pixels P, the neighborhood is thresholded using the pixel value, and the pixel value will be the sum of the binary number, and then after LBT histogram of regions is used to define the features. Chang et al. (2012) uses the LBT with the sMRI data fed as 2D images. The highest accuracy they achieved was 69.95%. Colby et al. (2012) applied SVM on features extracted from fMRI including pairwise Pearson's correlation, global graph theoretical metrics, nodal and global graph measures of the brain network, and morphological information from structural MRI including surface vertices, surface area, gray matter volume, average cortical thickness, etc. and 55% was achieved from the classification model. Dai et al. (2012) applied SVM on functional connections generated using fMRI data, and they achieved an accuracy of 65.87%. Itani et al. (2018) considered the statistical, frequency-based features extracted from resting-state fMRI data as well as demographic information and used the decision tree for classification. The highest accuracies they achieved were 68.3 and 82.4% for the sites New York and Peking, respectively. In Wang et al. (2016), the authors used KNN for the classification of functional connectivity generated using resting-state fMRI data processed using the maximum margin criterion, and the achieved accuracy was 79.7%. Eslami and Saeed (2018b) incorporated KNN as the classification method and used the EROS similarity measure for computing the similarity between the fMRI time series of different samples.

### 4.2. ASD Classification

Like ADHD, many studies applied traditional ML models for the classification of ASD. Our analysis indicates that many ML methods use ABIDE I/II as a gold standard data sets (Heinsfeld et al., 2018) to measure their classification accuracy. In Chen et al. (2016), authors used brain functional connectivity of different frequency bands as the features and applied SVM for the classification. In another work (Price et al., 2014), applied kernel support vector machine (MK-SVM) to static and dynamic functional connectivity features generated based on a sliding window mechanism. In Ghiassian et al. (2013), SVM is applied to a histogram of oriented gradients (HOG) features of fMRI data. The work presented in Katuwal et al. (2015) applied three different classification algorithms SVM, Random Forest (RF), and Gradient Boosting Machine (GBM) using sMRI. The highest accuracy across all sites was 67%. In another approach, Wang M. et al. (2019) used KNN and SVM as the classification method to the low-rank representation of fMRI data. The work presented in Chen et al. (2015) applied random forest to functional connectivity of different regions using fMRI data.

## 5. Detection of ASD/ADHD Using DL Methods

DL has become a popular tool for evaluating the utility of imaging in classifying those with and without different brain disorders. Countless studies focus on using deep neural networks for diagnosing ASD and ADHD. In the following subsections, we describe approaches that are designed based on deep or shallow neural networks and applied to MRI and fMRI data. These methods are used either as the classifier or as feature selectors/extractor.

### 5.1. ADHD Classification

Different shallow and deep neural network architectures have been proposed for ADHD classification. One of the first attempts to use a deep neural network is the study proposed by Kuang and He (2014). In their proposed method, fMRI time series of each voxel of the brain is transformed from the time domain to frequency domain using Fast Fourier transformation. Frequency associated with the highest amplitude is selected as the feature of each voxel and used for training a Deep Belief network. Deshpande et al. (2015) used Fully Connected Cascade neural network architecture applied to different variety of features generated from fMRI time series, such as pairwise Pearson's correlations, Correlation-Purged Granger Causality, the correlation between probabilities of recurrences and Kernel Granger causality. Hao et al. (2015) proposed a method called Deep Bayesian Network. Their method includes reducing the dimensionality of fMRI data by using the FFT and Deep Belief Network applied to each region of the brain, followed by constructing a Bayesian Network to compute the relationships between different brain areas and finally use SVM for the classification. Convolutional Neural Network is explored in multiple studies (Zou et al., 2017; Qu et al., 2019; Wang Z. et al., 2019). For example in the study proposed by Qu et al. (2019), the 3D kernel in convolutional network is replaced by their proposed 3D Dense Separated Convolution module in order to reduce the redundancy of 3D kernels.

### 5.2. ASD Classification

Different varieties of Autoencoders, such as shallow, deep, sparse, and denoising are widely used for extracting lower-dimensional features from fMRI (Guo et al., 2017; Dekhil et al., 2018b; Heinsfeld et al., 2018; Li H. et al., 2018; Eslami and Saeed, 2019; Eslami et al., 2019; Wang et al., 2019a) and sMRI (Sen et al., 2018; Xiao et al., 2018; Kong et al., 2019) data. Dvornek et al. (2017, 2018, 2019) explored the power of RNN and LSTM for analyzing fMRI data. In one of their proposed architectures, the output of each repeating cell in the LSTM network is connected to a single node making a dense layer (Dvornek et al., 2017). The averaged output of these nodes over the whole sequence is fed to a Sigmoid function and shows the probability of an individual having a diagnosis of ASD. In another study (Dvornek et al., 2018), authors expanded the previous method and incorporated phenotypic information to the proposed method. They investigated 6 different approaches, such as repeating phenotypic information along the time dimension, concatenating it to the time series and feeding it to the network, or feeding the phenotypic data and the final output of LSTM to the dense layer. CNN networks are also used in different studies for diagnosing autism (Brown et al., 2018; Khosla et al., 2018; Li G. et al., 2018; Parisot et al., 2018; Anirudh and Thiagarajan, 2019; El-Gazzar et al., 2019a,b). Khosla et al. (2018) proposed a multi-channel CNN network in which each channel represents the connectivity of each voxel with specific regions of interest. Their CNN architecture is made of several convolutional, max-pooling, and densely connected layers. Their proposed method is trained on different atlases separately and the majority vote of the models is used as the final decision. In another work based on CNN, El-Gazzar et al. (2019a) proposed using a 1D CNN which takes a matrix containing the average time series of different regions as the input. Their motivation behind this approach is using original time series as the input of the model instead of connectivity features proposed by other studies, hence extracting non-linear patterns from original time series data. Parisot et al. (2018) formulated the autism classification as a graph labeling problem. They represented the population of the subjects as a sparse weighted graph in which nodes represent the imaging features and phenotypic information is integrated as edge weights. The population graph is then fed into a graph convolutional neural network which is trained in a semi-supervised manner. Anirudh and Thiagarajan (2019) proposed bootstrapping graph convolutional networks for autism classification. In their proposed methods they followed the strategy proposed by Parisot et al. (2018) to construct the population graph. They generated an ensemble of population graphs and a graph CNN for each of them which is considered as a weak learner. Finally, the mean of the predictions to each class by all learners is computed and the label associated with the larger value is assigned to the test sample. Other variants of neural networks, such as Probabilistic neural networks, competition neural network, Learning vector quantization neural network, Elman neural network, etc are also used for ASD classification. Iidaka (2015) used a probabilistic neural network for training thresholded functional connectivity (pairwise Pearson's correlation) between fMRI time series extracted from different brain regions which achieved a high classification accuracy of 90%. Bi et al. (2018) used a cluster of neural networks containing Probabilistic neural networks, competition neural networks, learning vector quantization neural network, Elman neural network, and backpropagation neural network. The features they considered for their proposed methods consist of pairwise Pearson's correlation coefficient as well as graph-theoretical measures, such as degree, shortest path, clustering coefficient, and local efficiency of each brain network.

Since different settings are used for conducting the experiments, a direct comparison of these methods is not possible. Leave-one-out-cross validation and k-fold cross-validation (with *k* = 5 and 10) are the most frequently used evaluation methods in ASD analysis. On the other hand, the train-test split is more often used for ADHD analysis since the ADHD-200 consortium provided the predefined sets of train and test data. With that said, limitations inherent to the ADHD-200 dataset (Zhao and Castellanos, 2016; Wang et al., 2017; Zhou et al., 2019) as well as the collection of additional neuroimaging data across various research groups with smaller samples may result in increased adoption of leave-one-out-cross validation and k-fold cross-validation techniques in ADHD samples. Among traditional ML methods, SVM is the most frequently used traditional ML and CNN and Autoencoders are the most used DL methods. Most studies are carried out by using fMRI data. Even though the combination of fMRI and sMRI could be a much richer source of information, it has been used in fewer studies compared to using each modality separately.

**Figure 4:**
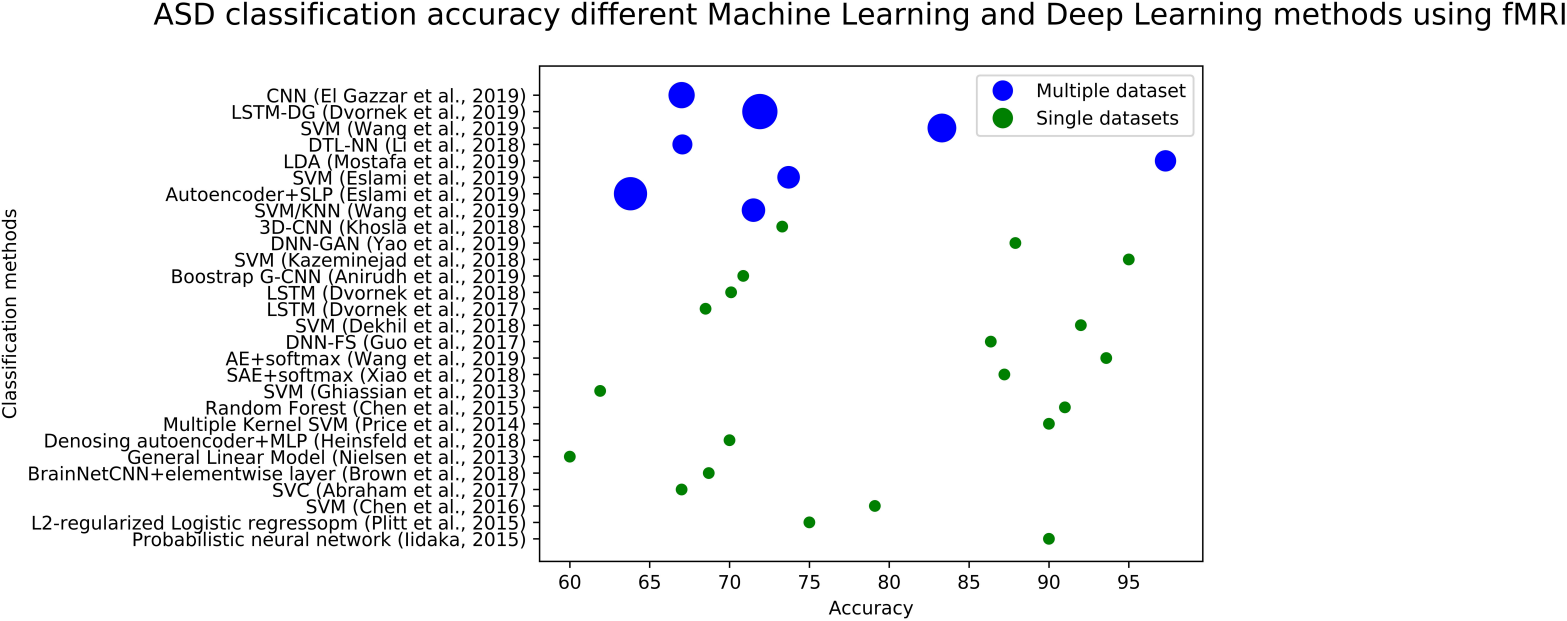
The graph shows fMRI based studies, to date, and their associated accuracy for classification of ASD. Single data sets refers to the accuracy reported by using data from a single site, and multiple data sets refers to accuracy reported in the paper using multiple sites.

**Figure 5:**
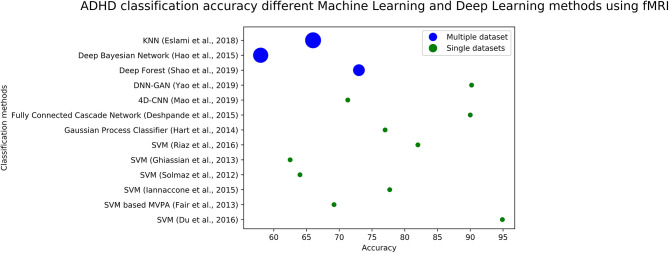
The graph shows fMRI based studies, to date, and their associated accuracy for classification of ADHD. Single data sets refers to the accuracy reported by using data from a single site, and multiple data sets refers to using multiple sites.

We plotted the accuracy of different methods using fMRI data in Figure 4 (for ASD diagnosis) and Figure 5 (for ADHD diagnosis). Each circle in each image corresponds to the accuracy reported in a study. Blue circles correspond to the methods that are tested on a single dataset, while green circles correspond to models that are evaluated on several datasets. The size of each blue circle indicates the standard deviation of accuracies over multiple datasets. Evaluating a model on multiple datasets provides a more realistic image of its generalizability. Even though the accuracy of the model could be very high on a single dataset, it may not necessarily perform the same across other datasets. Detailed information (including reported accuracy, Training size, Test size, and type of testing) related to ML/DL methods for diagnosing ASD using ML and DL methods with various modalities is listed in Table 1. Similar information about the ML/DL methods for diagnosing ADHD is listed in Table 2.

**Table 1:** Literature review of ASD diagnosis using ML and DL methods is shown.

**Modality**	**Train size**	**Test size**	**Classification method**	**Test type**	**Accuracy (%)**	**Remark**
fMRI	640		Probabilistic neural network (Iidaka, 2015)	LOOCV	90	Subjects are below the age of 20
	296		L2-regularized logistic regression (Plitt et al., 2015)	LOOCV	75	Age and IQ matched subjects
	240		SVM (Chen et al., 2016)	LOOCV	79.17	Subset of ABIDE 12–18 years old
	871		SVC (Abraham et al., 2017)	LOSO	67	
	774		3D-CNN (Khosla et al., 2018)	10-fold CV	73.3	
	1,013		BrainNetCNN+elementwise layer (Brown et al., 2018)	5-fold CV	68.7	
	964		General Linear Model (Nielsen et al., 2013)	LOOCV	60	
	1,035		Denoising AE+MLP (Heinsfeld et al., 2018)	10-fold CV	70	
	60		Multiple Kernel SVM (Price et al., 2014)	LOOCV	90	Subset of NYU dataset
	200	52	Random Forest (Chen et al., 2015)	train-test	91	Subset of ABIDE dataset
	888	222	SVM (Ghiassian et al., 2013)	train-test	61.9	
	93 ± 40		SVM/KNN (Wang M. et al., 2019)	5-fold CV	71.5 ± 4	
	60.8 ± 30.8		Autoencoder+SLP (Eslami et al., 2019)	10-fold CV	63.8 ± 8	
	92 ± 54		SVM (Eslami and Saeed, 2019)	5-fold CV	73.7 ± 3.7	
	49 ± 26		LDA (Mostafa et al., 2019)	10-fold CV	97.3 ±3.3	
	184		3DCNN C-LSTM (El-Gazzar et al., 2019b)	5-fold CV	77, 73	Two sites (NYU, UM) from ABIDE dataset
	77 ± 21		deep transfer learning neural network (DTL-NN) (Li H. et al., 2018)	5-fold CV	67.05 ± 2.9	
	48.2 ± 42.3		SVM (Wang et al., 2019b)	10-fold CV	83.3 ± 6	
	84		SAE + softmax (Xiao et al., 2018)	Averaged CV^*a*^	87.21	School aged children from ABIDE dataset
	1,054		AE + softmax (Wang et al., 2019a)	10-fold CV	93.59	
	110		DNN-FS (Guo et al., 2017)^*b*^	5-fold CV	86.36	Site UM from ABIDE-I
	283		SVM (Dekhil et al., 2018b)	LOSO^c^	92	Subject from NDAR^d^
	1,100		LSTM (Dvornek et al., 2017)	10-fold CV	68.5	
	1,100		LSTM (Dvornek et al., 2018)	10-fold CV	70.1	
	131 ± 34		LSTM-DG (Dvornek et al., 2019)	10-fold CV	71.9 ± 9	
	872		Boostrap G-CNN (Anirudh and Thiagarajan, 2019)	10-fold CV	70.86	
	51		SVM (Kazeminejad and Sotero, 2018)	10-fold CV	95	Subjects above the age of 30
	454		DNN-GAN (Yao and Lu, 2019)	5-fold CV	87.9	
	48 ± 27		CNN (El-Gazzar et al., 2019a)	LSOS^e^	67 ± 5	
sMRI	48.9 ± 31		Random Forest (Katuwal et al., 2015)	CV10-20	79 ± 9	
	182		Sparse stacked autoencoders + softmax (Kong et al., 2019)	LOOCV	90.3	NYU dataset from ABIDE
	132		SVM (Zheng et al., 2019)	LOOCV	78.63	
	276		Data expanding multi-channel CNN (Li G. et al., 2018)	10-fold CV	76.2	Subjects from NDAR
	64		SVM (Chaddad et al., 2017)	10-fold CV	67.85	Two sites (UM, Pitt) from ABIDE dataset
	650		SVM/KNN (Demirhan, 2018)	5-fold CV	52 ± 7	Subjects below 10 years are excluded
	44		SVM (Ecker et al., 2010)	LOOCV	77	
	734		Random Forest (Katuwal et al., 2015)	LOOCV	60	
	138	47	Projection Based Learning (PBL) (Vigneshwaran et al., 2013)	train-test	70	NYU dataset from ABIDE-I
	85		Random Forest (Xiao et al., 2017)	3-fold CV	80.9 ± 1.5	
	40		Projection Based Learning (PBL) (Subbaraju et al., 2015)	5-fold CV	98.67 ± 1.7	Subjects are only adult female
	78		SVM (Chen et al., 2020a)	10-fold CV	74	NYU dataset from ABIDE-II
	142		SVM (Yassin et al., 2020)	10-fold CV	89.6	36 ASD, and 106 TD
	38		Logistic Model Trees (LMTs) (Jiao et al., 2010)	10-fold CV	87	
fMRI + sMRI	871		Graph Convolutional Networks (Parisot et al., 2018)	10-fold CV	70.4	
	800	311	SVM (Sen et al., 2018)	train-test	64.3	
	47		DFCN (Dekhil et al., 2017)		94.7	Subjects from NDAR
	185		DBN (Aghdam et al., 2018)	10-fold CV	65.56	subjects in range 5–10 from ABIDE-I/II
	817		MLP (Rakić et al., 2020)	10-fold CV	85	
	809		Multichannel DANN (Niu et al., 2020)	10-fold CV	73.2	

a *Average result of 7, 14, 21, 28, 42, and 84 fold cross validation*.

b *DNN with a novel feature selection method*.

c *Leave one subject out*.

d *National Database of Autism Research*.

e *Leave site out cross validation*.

*The table gives an overview of the modalities used, training, and test size of the data, classification model, type of test used for evaluation as well as accuracy reported by the authors. Remarks gives relevant information to put the accuracy and other results in context for fair comparisons across different studies*.

**Table 2:** Literature review of ADHD diagnosis using ML and DL methods is shown.

**Modality**	**Train size**	**Test size**	**Classification method**	**Test type**	**Accuracy (%)**	**Remarks**
fMRI	216		SVM (Du et al., 2016)	10-fold CV	94.9	Subset of ADHD-200
	156		SVM based MVPA^*a*^ (Fair et al., 2013)	LOOCV	69.2	Subset of ADHD dataset 3 group classification
	36		SVM (Iannaccone et al., 2015)	LOOCV	77.7	Subjects are between 12–16 years
	506		SVM (Solmaz et al., 2012)	LOOCV	64	Subset of ADHD-200
	769	171	SVM (Ghiassian et al., 2013)	Train-test	62.5	
	60		Gaussian Process Classifier (Hart et al., 2014)	LOOCV	77	Task based fMRI
	210/193	41/51	Decision tree (Itani et al., 2018)	train-test	68.3/82.4	NYU/Peking datasets from ADHD-200
	126 ± 63	28 ± 12	KNN (Eslami and Saeed, 2018b)	train-test	66 ± 11	
	1,177		fully connected cascade (FCC) (Deshpande et al., 2015)	LOOCV	90	
	135 ± 71	32 ± 15	Deep forest (Shao et al., 2019)	train-test	73 ± 6	Subset of ADHD-200
	128 ± 62	34 ± 16	Deep Bayesian Network (Hao et al., 2015)	Train-test	58 ± 10	Subset of ADHD-200
	626	162	4D CNN (Mao et al., 2019)	Train-test	71.3	
	487		DNN-GAN (Yao and Lu, 2019)	5-fold CV	90.2	
	222/48	41/25	DBN (Farzi et al., 2017)	Train-test	63.6/69.8	Tested on NYU/NeuroImage from ABIDE
	621		SVM (Riaz et al., 2016)	Train-test	82	Peking, KKI, NYU, and NI datasets from ADHD-200
sMRI	111	48	Hierarchical Extreme Learning Machine (Qureshi et al., 2016)	train-test	60.78	Subset of ADHD-200
	110		Extreme Learning Machine (Peng et al., 2013)	LOOCV	90.18	Subset of Peking dataset from ADHD-200
	36		SVM (Iannaccone et al., 2015)	LOOCV	61.1	
	78		SVM (Igual et al., 2012)	5-fold CV	72.48	
	68		SVM (Johnston et al., 2014)	LOOCV	93	
	436		SVM (Chang et al., 2012)	10-fold CV	69.9	Subset of ADHD-200
	770	171	Tensor boosting (Zhang et al., 2017)	Train-test	69	
	587		Dilated 3D-CNN (Wang Z. et al., 2019)	5-fold CV	76.6	Subset of ADHD-200
fMRI + sMRI	558	171	SVM (Sen et al., 2018)	5-fold CV	68.9	
	776	197	SVM (Colby et al., 2012)	Train-test	59	
	559	171	Multi-Modality 3D CNN (Zou et al., 2017)	Train-test	69.15	

a *Multivariate Pattern Analysis. The table gives an overview of the modalities used, training, and test size of the data, classification model, type of test used for evaluation as well as accuracy reported by the authors. Remarks gives relevant information to put the accuracy and other results in context for fair comparisons across different studies*.

## 6. Existing Strategies to Avoid Common Pitfalls

### 6.1. Existing Techniques to Avoid Overfitting

Overfitting is an inevitable issue in training deep neural networks on small datasets. Since the number of sMRI and fMRI samples available in ADHD and ASD repositories are not large enough for successfully training a deep neural network, researches adopted different approaches to make their proposed methods robust to overfitting. In Eslami and Saeed (2019) and Eslami et al. (2019), the authors proposed a data augmentation technique that applies Synthetic Minority Oversampling Technique (SMOTE) (Chawla et al., 2002) to the examples of ASD and control classes and increased the size of the training set by 2-folds. In another study, Dvornek et al. (2017) proposed a data augmentation method by cropping 10 sequences of length 90 from each time series randomly which increased the size of the dataset by a factor of 10. Similarly, Mao et al. (2019) utilized a clipping strategy which samples fMRI time series to fixed intervals. In the work presented by Yao and Lu (2019), based on the idea of GAN, a network called WGAN-C is proposed to augment brain functional data. Dropout and L1/L2 regularization are heavily used in DL structures to avoid overfitting (Dvornek et al., 2017; Brown et al., 2018; Khosla et al., 2018; Parisot et al., 2018; Anirudh and Thiagarajan, 2019). Feature selection is another solution for reducing overfitting. Techniques, such as Recursive Feature Elimination (RFE) (Katuwal et al., 2015; Wang et al., 2019a,b), F-score feature selection (Peng et al., 2013; Kong et al., 2019), and autoencoders (Guo et al., 2017; Dekhil et al., 2018b; Heinsfeld et al., 2018; Li H. et al., 2018; Sen et al., 2018; Xiao et al., 2018; Eslami et al., 2019; Kong et al., 2019; Wang et al., 2019a) are widely used for reducing the number of features.

### 6.2. Strategies to Deal With Imbalanced Datasets

Class-imbalance is very common in medical datasets such that patients often represent the minority class (Rahman and Davis, 2013). This is consistent with the substantially lower base rates of disorders, such as ADHD or ASD and can create substantial challenges related to optimizing accuracy by reducing both false positives and false negatives simultaneously (Youngstrom, 2014). Training ML models using imbalanced data makes the model biased toward the majority class. Class-imbalanced can be observed in ASD and ADHD datasets, especially in ADHD-200 which consists of 491 healthy and 285 ADHD subjects. Different approaches are utilized to reduce the effect of the majority class on the final prediction. The machine-learning community in general has addressed the issue of class-imbalance in two ways (Chawla et al., 2002): One is by assigning distinct costs to training examples, and the other is to re-sample the original data by either oversampling the smaller minority class or by under-sampling the larger majority class. There are several techniques to address imbalanced datasets in sMRI/fMRI data for ASD and ADHD classification. These include k-fold cross validation (Qureshi and Lee, 2016; Eslami et al., 2019) (randomly splitting the data while maintaining class distribution for k times), re-sampling training set (Colby et al., 2012; Li X. et al., 2018) (under-sampling or over-sampling training set to have an even class distribution), and bootstrapping (Beare et al., 2017; Dekhil et al., 2018a) (re-sampling the dataset randomly with replacement to oversample the dataset). One method for handling imbalanced data in ADHD, and ASD data sets is SMOTE which is used to oversample the minority class (Riaz et al., 2016; Farzi et al., 2017; Shao et al., 2019). SMOTE (Chawla et al., 2002) is a technique to adjust the class distribution of a data set, or to produce synthetic data for your ML model. SMOTE technique shows that a combination of over-sampling the minority class and under-sampling the majority class allows machine-learning, and deep-learning methods to achieve better classifier performance when compared with the performance of only using under-sampling the majority class. The performance is generally defined in the ROC space, and compared with the loss ratios than one would get from Ripper or Naïve Bayes. The authors have successfully used SMOTE technique on MRI data sets for ADHD and ASD classifications machine-learning models (Eslami and Saeed, 2018b, 2019; Eslami et al., 2019).

## 7. Frontiers and Future Direction in Machine-Learning for ASD and ADHD MRI Data Sets

Rapid improvement in machine-learning techniques will allow further breakthroughs in ADHD and ASD diagnosis that is based on imaging techniques. Here we highlight two directions which would be beneficial for taking forward the field of computational diagnosis of ADHD and ASD.

### 7.1. Extracting More Knowledge From Smaller Data Sets

One way to improve the performance of machine-learning and deep-learning techniques is to feed more data to the model to reduce overfitting and improve generalizability. However, MRI acquisition is time consuming and costly, and does not allow strict control of parameters needed for machine-learning algorithmic development. One cost-effective way to enhance generalization, increase reproducibility, and reliability of machine-learning models is to perform data augmentation using available training sets. Large datasets are a must-have when it comes to training deep neural networks in order to optimize the learning process. Data augmentation techniques (Shin et al., 2018) can be used to generate artificial data using available training data which is useful when data collection is costly or not possible. Augmenting data can be done in an online or offline fashion. In the former case, new data is generated before the training process is started and the model is trained using the pool of real and artificial data. This method is preferred for small datasets. In the latter, new data is generated in each batch feeding to the network. This method is preferred for large datasets. Flipping, translation, cropping, adding Gaussian noise, and blurring images are examples of popular data augmentation methods used in computer vision area.

### 7.2. Establishing Fundamental Principles for Autism and ADHD

Discovery of laws and scientific principles using machine-learning solutions is a transformative (albiet not new) concept in science. However, clinical scientists, mental health providers, and physicians are reticent to adopt artificial intelligence, often because of the lack of *interpretability*. Long-term vision for computational neuroscience is to address this issue by developing the necessary methodology to make ML and DL algorithms more transparent and trustworthy to these providers, particularly with respect to correctly classifying ADHD and ASD patients. ML interpretability can be used for many purposes: build trust, favorize acceptance, compensate unfair biases, diagnose how to improve models, and certify learned models. More importantly the interpretation of the models could help discover new knowledge that might be useful for neuroscientists for future studies (e.g., a specific neural pathway discovered for ADHD or ASD that is not known and found in neurotypical brains). As a result, ML and DL interpretability has become a core national concern when applied to biomedical decision making (see National AI R&D Strategic Plan: 2019), and will require significant efforts and resources. Investigations into frameworks that support knowledge discovery by using transparent ML/DL models that will encode the known underlying neurobiology, extract rules from neural networks, and translate that into actual neural pathways of the human brain would give us extraordinarily insights. Currently we are not aware of any interpretable deep-learning model for ADHD or ASD classifications.

### 7.3. Novel Methods to Integrate the Multimodality of MRI Data Sets

Due to the sparse nature of structural connectivity, most functional connections are not supported by an underlying structural connection. Community detection for structural and functional networks typically yield different solutions and models that can integrate feature-vectors to produce classification accuracy greater than both (sMRI and fMRI) models is challenging. One challenge is the distinct cardinality of feature vectors from two models that needs to be integrated to boost accuracy performance. The integration model must also be able to distinguish between feature that are instrumental in correct classification and reduce the effect of features which produce adverse results. Classification of neuroimaging data from multiple acquisition sites that have different scanner hardware, imaging protocols, operator characteristics, and site-specific variables makes efficient and correct integration of sMRI and fMRI data extremely challenging. To our knowledge, only one deep-learning technique (Zou et al., 2017) has been introduced for integration of fMRI and sMRI data sets which gives maximum accuracy of 69%. Provided that the neuroimaging markers identified from integration of sMRI/fMRI data must be reliable across imaging sites to be clinically useful. Since deep-learning is especially useful in identifying complex patterns in high-dimensional fMRI data; integrated methods that can deal with high dimensional sMRI/fMRI data, if designed correctly, must lead to high accuracy and more formal investigated is warranted. We are not aware of a deep-learning model that allows such integration that provides higher accuracy comparing to current state-of-the-art fMRI/sMRI based methods.

### 7.4. High Performance Computing Strategies

Current machine-learning (especially deep-learning) approaches are too slow and thus detracting from making appropriate gains in classification of psychiatric biomarkers. Recent proliferation of “big data” and increased calls for data sharing particularly with fMRI data will necessitate novel approaches that have the ability to quickly and efficiently analyze this data to identify appropriate biomarkers of ADHD and ASD.

Carefully crafted parallel algorithms that take into account the CPU-GPU or CPU-Accelerator architecture are critical for scalable solutions for multidimensional fMRI data. Future HPC strategies would requires two components for scalable frameworks: (1) parallel processing of MRI data and (2) parallel processing of deep-learning networks that are associated with ASD and ADHD diagnosis. Although rarely employed till date, few HPC methods specific to MRI data analysis has been proposed (Eslami et al., 2017; Tahmassebi et al., 2017; Eslami and Saeed, 2018a; Lusher et al., 2018).

## 8. Discussion

Despite being at the early stages, Machine-Learning (ML), and Deep-Learning (DL) methods have shown promising results in diagnosing ADHD and ASD in most cases. DL models are overtaking traditional models for feature extraction and classification. Although DL can provide accurate decisions, there are several challenges that need to be considered while using it. DL methods were not specifically designed for neuroimaging data which usually contains a small number of samples and many features leading to overfitting (Jollans et al., 2019). Avoiding overfitting has become the focus of recent studies that use solutions, such as dropout, regularization, and data augmentation. Another issue with DL methods is the lack of transparency and insight which makes them known as *black boxes*. Even though the structure of the network is explainable, they are not able to answer the questions like why the set of provided features used in the training provides the network predictions, or what makes one model superior to another one. *Interpretability* is an important factor for trusting such models, which is a necessity for understanding brain abnormalities and differences between controls and patients. This aspect is missing from most of the designed architectures and is an area that needs more focus and attention. Finally, integration of research findings from the ML and DL literature as well as adoption of the use of such approaches in combination with neuroimaging data by practitioners in everyday clinical practice are likely to be met with some resistance given the limitations noted above among others. For example, neuroimaging is costly and involves substantial time commitments by multiple individuals (e.g., MRI technicians, physicians, patients) that are currently not involved typically in the diagnosis of ADHD or ASD. As a result, the extent to which data collected via neuroimaging is likely to aid clinicians in practice will depend largely upon whether machine learning algorithms applied to such data are more optimal at classifying those with and without the disorder relative to more traditional methods (e.g., rating scales, interviews) requiring fewer resources (e.g., shorter time duration, more cost effective), and are explainable.

One aspect that directly affects the accuracy of the model is the distribution of the training data which should be representative of the unseen data. Public brain imaging datasets, such as ADHD-200 and ABIDE gathered the data from several brain imaging centers in different geographical locations in which different scanners, scanning settings, and protocols are used for generating images. These differences can affect the distribution of the data and deteriorate the ability of the model to perform correct predictions for other samples. This aspect is mostly overlooked in analyzing the performance of the proposed models which may focus on a subset of these benchmarking datasets such that performance of the model on other datasets is unclear. To reflect the realistic performance of an ML model in diagnosing brain disorders, these models must be tested on multiple datasets to guarantee generalizability. Using the same validation process among different studies also ensures the fairground for comparisons and reproducible benchmarking. The reproducibility of ML methods is another important concept that should be considered. Designing an ML/DL model consists of many details about the hyperparameters and training process, such as number of layers, number of nodes in each layer, number of iterations, hyperparameter tuning methods, regularization methods used for avoiding overfitting, types of activation functions, types of loss functions, etc. Unless making the implementation of the model available to the public, or providing all the details used for implementation, reconstructing the same model and getting the same result is not possible. Sharing the implementation of the model along with proper guidelines for using it makes the process of reproducing the results a better experience for other researchers. The scientific codes for these methods should be re-runnable, repeatable, reproducible, reusable and replicable (Benureau and Rougier, 2018). Recently proposed schemes, such as—the Brain Imaging Data Structure (BIDS) (Gorgolewski et al., 2016)—will standardize data organization, storing, and curation processes which will streamline reliability and reproducibility of the machine-learning, and deep-learning models.

There is still room for improving the current research studies to provide a better diagnostic experience. One issue that is overlooked by most research studies is the running time needed for training the predictive models. As mentioned earlier, ML and DL methods are not originally designed for brain imaging data. For example, CNN model is initially designed for classifying 2D images, however, in MRI and fMRI we are dealing with 3D and 4D data. Extending the original CNN architecture from 2D to 3D and 4D increases the number of parameters and overall running time. The long running time could be a hurdle for a tool assisting in medical diagnosis and high-performance computing algorithms could be vital to make these ML model mainstream. fMRI and sMRI features are mostly considered individually as predictors to ML models, while their combination can provide a richer source of information. Using the fusion of sMRI and fMRI data, particularly when combined with other information (e.g., demographic characteristics) could be a potential way to further improve the predictability and interpretability of the ML models. There is room for improving the quality of predictive models by employing data augmentation and transfer learning methods. The success of these methodologies in other fields, such as computer vision encourages incorporating them in designing predictive models for diagnosing brain disorders.

## Author Contributions

TE and FS conceived and designed the study. TE did the implementation of the code and results. TE, FA, JR, and FS interpreted the results and wrote the manuscript. FA and FS read and synthesized sMRI knowledge specific to ASD and ADHD classification, and interpreted the results. All authors contributed to the article and approved the submitted version.

## Conflict of Interest

The authors declare that the research was conducted in the absence of any commercial or financial relationships that could be construed as a potential conflict of interest.
