# Supplementary material for: Machine Learning Methods for Diagnosing Autism Spectrum Disorder and Attention- Deficit/Hyperactivity Disorder Using Functional and Structural MRI: A Survey
Source: Front Neuroinform. 2021 Jan 20;14:575999. doi: 10.3389/fninf.2020.575999 (PMC7855595; doi:10.3389/fninf.2020.575999)
Supplement: Supplementary file 1 [file Data_Sheet_1.zip › supplementary/Supplementary_information.pdf]

Supplementary information

Taban Eslami[1], Fahad Almuqhim[3], Joseph S. Raiker[2], and Fahad Saeed[3,*]

[1]Department of Computer Science, Western Michigan University, Kalamazoo, USA
[2]Department of Psychology, Florida International University, Miami, USA
[3]School of Computing and Information Sciences, Florida International University, Miami, USA
[*]e-mail: fsaeed@fiu.edu

1 Overview of ML and DL methods

In the following subsections, we briefly describe the common ML and DL techniques.

1.1 Support Vector Machines

Support Vector Machines (SVM), also known as the maximum margin classifier, is undoubtedly one of the most popular ML techniques. The idea of SVM is to maximize the distance (margin) between the decision boundary (separating hyper-plane) and the closest samples of each class[1]. SVM can handle linearly separable classes, however, kernel trick is used to deal with non-linear classification. In this method, a kernel function is used to map the data points to a higher dimensional space in which they are linearly separable. Radial Basis Function (RBF), Polynomial, and Gaussian are examples of famous kernel functions.

1.2 Decision tree and Random Forest

A decision tree is an interpretable ML model that classifies samples based on a series of decisions. Each decision splits the data based on the most informative feature. Starting from the root of the tree and considering all features, this process is repeated until a user defined condition is met, for example, the height of the tree exceeds or the number of samples per each node is below a predefined threshold. Decision trees are prone to overfitting which can be avoided using techniques such as pre-pruning and post-prunning[2].

Random forest is a popular ML method that is considered as an ensemble of Decision Trees and helps to reduce the overfitting. In random forest, several trees are generated by randomly selecting samples and features for each tree, and after aggregating the predictions, the class label with the majority vote is used as the final prediction by random forest.

1.3 Naive Bayes classifier

Naive Bayes is a commonly used probabilistic classification technique based on the idea of Bayes' theorem. The term naive is based on the assumption of independence among features in a dataset that is violated for most of real world examples. In Naive Bayes classifier, given a sample with feature vector $X = x_1, x_2, ..., x_d$, the probability of a sample belonging to class k, ($P(C_k)$) is computed using the following equation:

$$P(C_k|X) = \frac{P(X|C_k)P(C_k)}{P(X)}$$

In which $P(C_k|X)$, $P(X|C_k)$, $P(C_k)$ and $P(X)$ are called *posterior probability*, *conditional probability*, *prior probability* and *evidence* respectively. Based on the independence assumption among features, conditional probability can be computed using the following equation:

$$P(X|C_k) = P(x_1|C_k)P(x_2|C_k)\dots P(x_d|C_k) = \prod_{i=1}^{d} P(x_i|C_k) \tag{1}$$

After computing the posterior probability of each class, the class with the highest probability will be considered as the prediction.

1.4 Autoencoder

Autoencoder is a specific type of feed forward neural network with the goal of reconstructing the input data in the output as close as possible. An autoencoder has the same number of nodes in the input and output layer and comprises of one or more hidden layers. In order to avoid the network to learn trivial functions such as identity function, usually the size of hidden layers are considered smaller than the size of the input (and output) layer. This forces the network to compress the data into smaller latent space ($h = g(x)$) which is called the encoding process. Next, in decoding process, the latent representations h, which

contain important patterns about the data, is used to reconstruct the original input ($x = f(h) = f(g(x))$). The structure of an autoencoder is shown in figure 1 (A). There are different extensions of the classic Autoencoder such as denoising Autoencoder and variational autoencoder. Denoising Autoendoer corrupts the data purposely by randomly turning some of the input values to zero. This process forces the network to learn more robust features and avoids learning the identity function by trying to reconstruct the uncorrupted version of the data. The idea of Variational Autoencoder (VAE) is quite different from traditional and denoising autoencoders. VAE is a generative model that instead of mapping the input data a vector of latent space, maps it to a distribution (vectors of the mean and standard deviation of the distribution). A variation of the input can be generated by randomly sampling from the distribution learned in the latent space. figure 1 (B) shows the structure of a VAE.

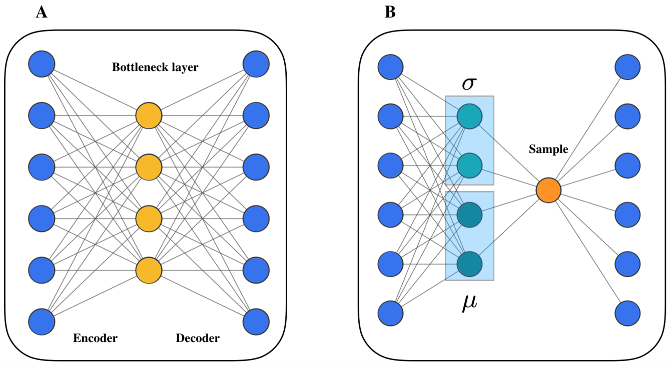

Figure 1. Structure of a classic autoencoder (A) and Variational Autoencoder (B).

1.4.1 *Convolutional Neural Network*

Convolutional Neural Network (CNN) is a very well used DL model in the computer vision area which is inspired by the visual cortex of the human brain. CNN is able to extract high level features in the last layers of the network by using lower level features provided in earlier layers. For example, in face images, low level features are the edges that are extracted from earlier layers and high level features are face components that can be extracted by combining lower level features. CNN networks are composed of three types of layers, the first type is *convolution* layer. The convolution layer is responsible for performing the convolution operation which extracts the spatial features from the input data. In the context of CNN networks, the convolution operation can be simply considered as the elementwise dot product of two matrices. In the case of 2D images, convolution is performed between a matrix called kernel (or filter) and a patch of an input image. The kernel has the same number of dimensions but smaller size than the input image which contains learnable parameters that should be optimized during the training process. After convolving with a patch of input data, the kernel shifts to the next patch and performs the same operation. The number of units shifted by the kernel is called stride which is a hyperparameter of the convolutional layer. The extracted features by the convolutional layer form another matrix called feature map, which is the input of the next layer called *pooling*. The pooling layer is responsible for reducing the number of parameters of the network, which in turn reduces the computation time as well as the chance of overfitting. Finally a *fully connected* layer will compute the class scores based on the features generated using previous layers. Figure 2 shows the architecture of a CNN.

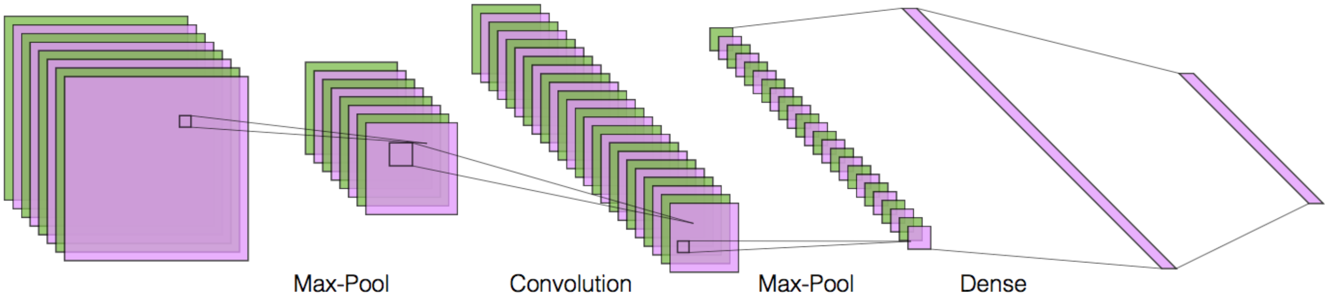

Figure 2. Structure of a Convolutional Neural Network

1.5 Recurrent Neural Networks

Recurrent Neural Network (RNN) is a special type of neural network that is designed for modeling sequence data by remembering past information. Similar to feed forward networks, RNN consists of input, hidden and output layers, with the difference that the hidden layer in RNN, receives its input from its previous layer at the time step t as well as the values of the same hidden layer in the previous time step $t-1$. This property provides this type of network the ability to remember previous information in a sequence, or in other words, provides a memory for the network. The architecture of RNN is shown in figure 3

RNN includes three categories for performing different sequence modeling tasks: one-to-many, many-to-one and many-to-many. In the one-to-many category, the input is a non-sequence while the output generates a sequence, like an image captioning task. In Many-to-one group, the input is a sequence while the output is a non-sequence task, for example, classification of a text review in sentiment analysis. Many-to-many includes the tasks in which both input and output represent a sequence, such as translating a sentence from one language to another. RNNs suffer from problems such as vanishing or exploding gradient which makes them unable to handle long term dependencies. These problems are due to the fact that during the back-propagation process for training, depending on the activation function used in the network, multiplication of continuous gradients may decrease or increase exponentially. In vanishing gradient, the gradients of earlier layers will get values close to zero, which doesn't allow them to contribute to the result.

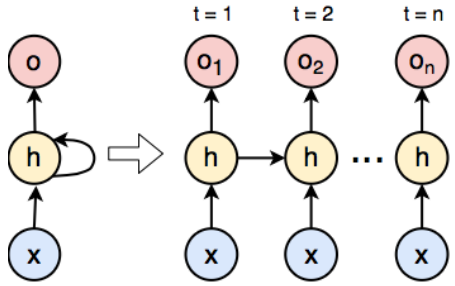

Figure 3. A) structure of a Recurrent Neural Network

1.6 Generative Adversarial Network

Generative Adversarial Network (GAN) proposed by Goodflow et al[3] is a generative model designed to generate synthetic data with the same distribution as its training dataset. GAN consists of two networks, Generative (G) and Discriminative (D) which work in an adversarial mode such that G tries to generate fake new data by learning the distribution of training data and fool the D, while D tries to detect fake data from real data. Figure 4 depicts the concept of GAN. Based on the success of GAN in different areas like data augmentation and image to image translation, it has rapidly adopted in the medical imaging domain for tasks such as image reconstruction, segmentation, and classification.

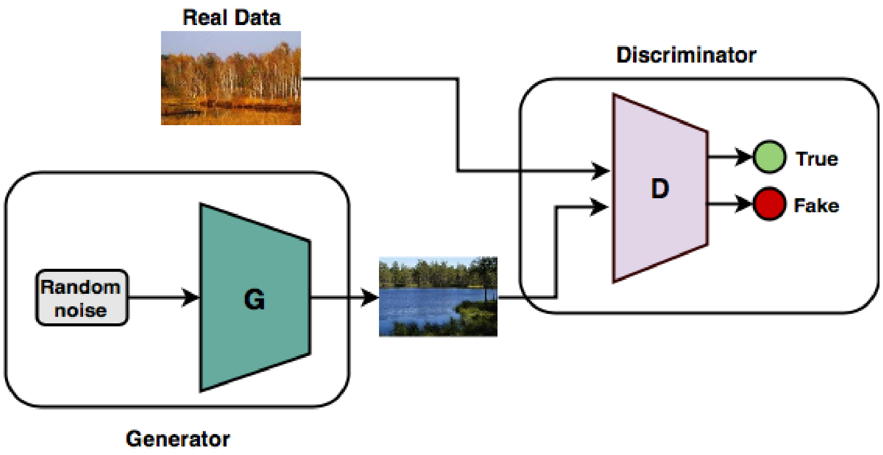

Figure 4. Generative Adversarial Network Structure

1.7 Other types of networks

There is a wide variety of networks designed by modifying the well known architectures we explained so far. An example is the family of graph neural networks. In many applications data comes in the form of complex graphs. The dependency and complexity existing in graph data make applying deep neural networks difficult to them[4]. Recently, deep neural network approaches are expanded to work with graph data, resulting in more generalized architectures such as graph convolutional neural network, graph Autoencoders, and graph recurrent neural networks. Lots of other famous architectures can be found in computer vision literature, such as AlexNet[5], GoogleNet[6], VGG Net[7], ResNet[8], U-net[9] and etc.

2 Public Autism and ADHD datasets

Neuroimaging data sharing has provided large-scale brain imaging datasets collected across laboratories and hospitals around the world to the public. These datasets offer researchers an opportunity to conduct large scale data-driven analysis to understand the cause and diagnose brain disorders. Here, we introduce the widely used public repositories for ADHD and ASD which provide brain imaging data including fMRI and sMRI along with other demographic information for subjects.

2.1 ADHD repositories

The most widely used brain imaging dataset for diagnosing ADHD is ADHD-200. This dataset was released in 2011 as a part of the ADHD-200 competition. The goal of this competition was to identify ADHD biomarkers using available data. This dataset contains a set of 947 samples (585 healthy and 362 ADHD) collected from 8 different brain imaging centers. Preprocessed sMRI and fMR as well as phenotypic information of each subject is provided in the dataset. The preprocessing steps used to preprocess MRI data include Skull-stripping, segmentation into CSF, WM, and GM, constructing WM and CS, computing a transformation between anatomical and template images, writing skull stripped anatomical image as well as GM probability image into template space at 1mm x 1mm x 1mm and Blur the normalized GM image. These steps are performed using AFNI and FSL software. fMRI prepossessing steps include Slice timing correction, motion correction, excluding non-brain voxels, co-registration to the corresponding anatomic image, Writing the data into template space at 4 mm x 4 mm x 4 mm and band-pass filtering.

2.2 ASD repositories

2.2.1 Autism Brain Imaging Data Exchange

One of the most used brain imaging datasets for ASD diagnosis is the Autism Brain Imaging Data Exchange (ABIDE). ABIDE contains two large collections of brain imaging and phenotypic data called ABIDE-I and ABIDE-II which is collected by 24 international brain imaging centers around the world. ABIDE-I dataset is collected by 17 different centers and contains 539 ASD and 573 healthy control samples released in 2012. The preprocessed version of ABIDE-I is performed by 5 teams using different preprocessing pipelines (the Connectome Computation System (CCS), the Configurable Pipeline for the Analysis of Connectomes (CPAC), the Data Processing Assistant for Resting-State fMRI (DPARSF) and the NeuroImaging Analysis Kit) and four different preprocessing strategies within each pipeline. Structural preprocessing was performed by three different pipelines (ANTS, CIVET, and FreeSurfer).

To provide more samples, the ABIDE II dataset released with 1114 additional samples (521 ASD and 592 controls) collected from 19 different sites around the world.

2.2.2 IMaging-PsychiAtry Challenge: predicting autism

IMPAC is a data challenge on detecting Autism that was held in 2018. A dataset containing MRI, resting-state fMRI and demographic information of 1150 public samples and 1003 test samples was released by the competition[10].

2.2.3 National Database for Autism Research (NDAR)

NDAR is a data repository containing heterogeneous Autism datasets. This dataset contains genomics, biomedical data as well as clinical and behavioral assessments. As of April 2015, NDAR contains the data of 117,573 subjects which include 4000 EEG and 2000 MRI images data[11]. Several studies used the subsets of data from this dataset for diagnosing ASD using ML methods[12–14].

3 Assessing the performance of the ML/DL models

One of the most important parts of designing ML methods is evaluating the performance of the model on unseen data to ensure the model is not suffering from underfitting or overfitting. The common techniques for evaluating ML models are the hold-out method and K-fold cross validation.

3.1 Hold-out Method

In this method, the dataset is split into two disjoint sets, the training set, and the test set. The training set is used for training the model and evaluating the hyperparameters. After the training process is completed, the model is tested on the holdout test set and the accuracy of the model is measured.

3.2 k-fold cross validation

In k-fold cross-validation, the whole data is divided into k disjoint sets. In each iteration $k-1$ sets are used for training and the remaining set is used for evaluating the model. This process is repeated k times and in each round, a different set is used as the test set while the others are used as training that ensures the model is evaluated on the whole samples of the dataset. At the end of this process, the performance of the model is considered as the average performance in each iteration of k-fold cross-validation.

Demonstration of k-fold cross-validation and hold-out paradigm is provided in figure 5.

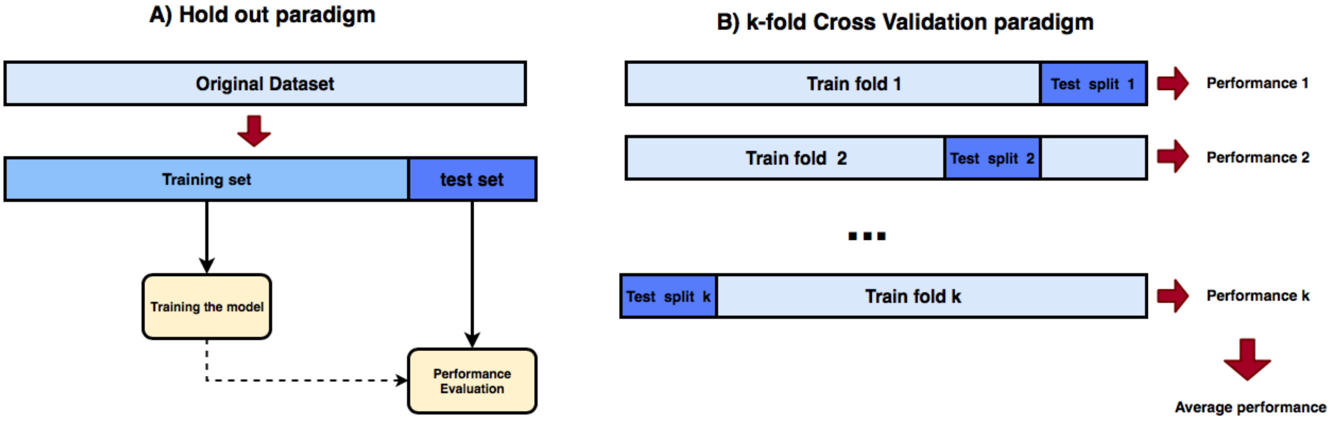

Figure 5. Hold-out paradigm vs k-fold cross-validation paradigm

4 Diagnostic Accuracy Measures

Diagnostic abilities of ML Models are evaluated by comparing the test result (the label they assign to a test sample) with the gold-standard status of the sample (actual label). In the case of binary classification, the model predicts a test sample to have the disorder (positive) or being healthy (negative) which divides the test population into 4 subgroups which can be shown in a confusion matrix as below:

As can be observed from the confusion matrix, **TP** refers to the number of patients which are correctly classified as patients by the model, **TN** refers to the number of healthy samples detected as healthy, **FP** refers to healthy samples which are incorrectly detected as patients and **FN** as the number of patients detected as healthy. The concept of negative of positive as healthy and patients may be used interchangeably among different studies. The most popular measures for evaluating the performance of a classifier can be derived from a confusion matrix as below.

Figure 6. Confusion matrix

		**Actual Values**	
		(+)	(−)
**Predicted Values**	(+)	TP	FP
	(−)	FN	TN

Accuracy of a classifier is defined as the ratio of correctly classified samples and can be computed using the following equation.

$$\frac{TP+TN}{TP+TN+FP+FN} \tag{2}$$

Sensitivity of the model which is also known as recall, is defined as the proportion of correctly classified patients.

$$\frac{TP}{TP+FN} \tag{3}$$

Specificity of the model defined as the proportion of correctly classified healthy subjects.

$$\frac{TN}{TN+FP} \tag{4}$$

Positive Predictive Value, also known as precision, shows the probability that the subject has the disorder when the test result is positive.

$$\frac{TP}{TP+FP} \tag{5}$$

Negative Predictive Value shows the probability that the subject has the disorder when the test result is negative.

$$\frac{TN}{TN+FN} \tag{6}$$

Table 1. *Graph theoretical properties*

Measure	Equation
Degree distribution	$k_i = \sum_{i \in n} a_i$<br>a_i: degree of node i
Closeness	$C(x) = \frac{1}{\sum_y d(x,y)}$<br>$d(x,y)$: Distance between vertices x,y
Betweenness	$g(v) = \sum_{s \neq v \neq t} \frac{\sigma_{st}(v)}{\sigma_{st}}$<br>$\sigma_{st}$: the number of shortest maths from s to t<br>$\sigma_{st}(v)$: the number of paths in σ_{st} that passes node v
Eigenvector centrality	$x_t = \frac{1}{\sigma} \sum_{t \in G} a_{v,t} x_t$
Transitivity	$T = \frac{\sum_{i \in n} 2t_i}{\sum_{i \in n} k_i(k_i - 1)}$
Local clustering coefficient	$T = \frac{2\lvert\{e_{jk} : v_j, v_k \in N_i, e_{jk} \in E\}\rvert}{k_i(k_i - 1)}$<br>$k_i$: number of neighbors of vertex i<br>e_{jk} : the edge between vertices $v_i$and v_j
Global clustering coefficient	$C = \frac{1}{n} \sum_{i=1}^{n} C_i$<br>$C_i$: Local clustering coefficient of node i

Another useful tool for evaluating the diagnostic ability of a classifier is **Receiver Operating Characteristic** graph. This tool can be used along with the classifiers which can return the probability of a sample belonging to class healthy or patient. For this type of classifiers, the final decision is based on comparing this probability with a predefined cut-off threshold. ROC curve plots the **TPR** (sensitivity) on the y-axis and **FPR** (1-specificity) on the x-axis by shifting the value of the threshold from 0 to 1. The area under this curve is called (**AUC**) and shows the ability of the model in distinguishing between the class of healthy and patients.

5 Graph theoretical measures

Table 1 shows the list of graph centrality measures that can be used for extracting features from brain functional network and fed to ML and DL methods.

6 Pros and cons of DL methods

Pros and cons of ML and DL methods explained above are provided in Table 2 and 3.

Table 2. Pros and cons of different DL methods

Model	Category	Learning Category	Pros	Cons
DNN	Discriminative	Supervised	• Detects complex non-linear relationships<br>• Widely used	• Computationally expensive<br>• Prone to overfitting<br>• Does not work with time series
CNN	Discriminative	Supervised	• Scale and shift invariant<br>• Reduced number of parameters	• Computationally expensive<br>• Prone to overfitting
RNN	Discriminative	Supervised	• Keeps short term information in sequence data	• Unable to retain long-term information in sequence data<br>• Computationally expensive
AE	Generative	Unsupervised	• Removes unnecessary information and noise	• Unable to preserve sparse representation<br>• Computationally expensive
GAN	Generative<br>Discriminative	Supervised<br>Unsupervised	• Removes unnecessary information and noise	• Unable to preserve sparse representation<br>• Hard to learn to generate discrete data like text

Table 3. Pros and cons of ML methods

Model	Pros	Cons
Support Vector Machines	• Can Model non-linear decision boundaries using kernel trick<br>• Performs well in high dimensional space	• Computationally expensive<br>• Difficult to select suitable kernel
Decision Tree	• Easy to interpret<br>• Can work with numerical and categorical features	• Prone to overfitting
Random Forest	• Performs well with non-linear decision boundarries	• Computationally expensive<br>• Less interpretable than an individual decision tree
Naive Bayes	• Performs fast and is easy to interpret<br>• When independent assumption among features hold, performs very well.	• Assumption of independence among features is usually not hold
K Nearest Neighbor	• Simple algorithm with no assumption about the data<br>• Easy to implement and performs relatively well	• Computationally expensive<br>• Optimal value of k and similarity measure is not trivial

References

1. Raschka, S. & Mirjalili, V. *Python Machine Learning, 3rd Ed.* (Packt Publishing, Birmingham, UK, 2019).
2. Duchesnay, E. *et al.* Feature selection and classification of imbalanced datasets: application to pet images of children with autistic spectrum disorders. *Neuroimage* **57**, 1003–1014 (2011).
3. Goodfellow, I. *et al.* Generative adversarial nets. In *Advances in neural information processing systems*, 2672–2680 (2014).
4. Wu, Z. *et al.* A comprehensive survey on graph neural networks. *arXiv preprint arXiv:1901.00596* (2019).
5. Krizhevsky, A., Sutskever, I. & Hinton, G. E. Imagenet classification with deep convolutional neural networks. In *Advances in neural information processing systems*, 1097–1105 (2012).
6. Szegedy, C. *et al.* Going deeper with convolutions. In *Proceedings of the IEEE conference on computer vision and pattern recognition*, 1–9 (2015).
7. Simonyan, K. & Zisserman, A. Very deep convolutional networks for large-scale image recognition. *arXiv preprint arXiv:1409.1556* (2014).
8. He, K., Zhang, X., Ren, S. & Sun, J. Deep residual learning for image recognition. In *Proceedings of the IEEE conference on computer vision and pattern recognition*, 770–778 (2016).
9. Ronneberger, O., Fischer, P. & Brox, T. U-net: Convolutional networks for biomedical image segmentation. In *International Conference on Medical image computing and computer-assisted intervention*, 234–241 (Springer, 2015).

10. Toro, R. Impac imaging-psychiatry challenge: predicting autism a data challenge on autism spectrum disorder detection @ONLINE (2020).

11. Payakachat, N., Tilford, J. M. & Ungar, W. J. National database for autism research (ndar): big data opportunities for health services research and health technology assessment. *Pharmacoeconomics* **34**, 127–138 (2016).

12. Dekhil, O. *et al.* A novel cad system for autism diagnosis using structural and functional mri. In *2017 IEEE 14th International Symposium on Biomedical Imaging (ISBI 2017)*, 995–998 (IEEE, 2017).

13. Li, G., Liu, M., Sun, Q., Shen, D. & Wang, L. Early diagnosis of autism disease by multi-channel cnns. In *International Workshop on Machine Learning in Medical Imaging*, 303–309 (Springer, 2018).

14. Dekhil, O. *et al.* Using resting state functional mri to build a personalized autism diagnosis system. *PloS one* **13**, e0206351 (2018).
