# Supplementary figures and images for: Machine Learning Methods for Diagnosing Autism Spectrum Disorder and Attention- Deficit/Hyperactivity Disorder Using Functional and Structural MRI: A Survey
Source: Front Neuroinform. 2021 Jan 20;14:575999. doi: 10.3389/fninf.2020.575999 (PMC7855595; doi:10.3389/fninf.2020.575999)

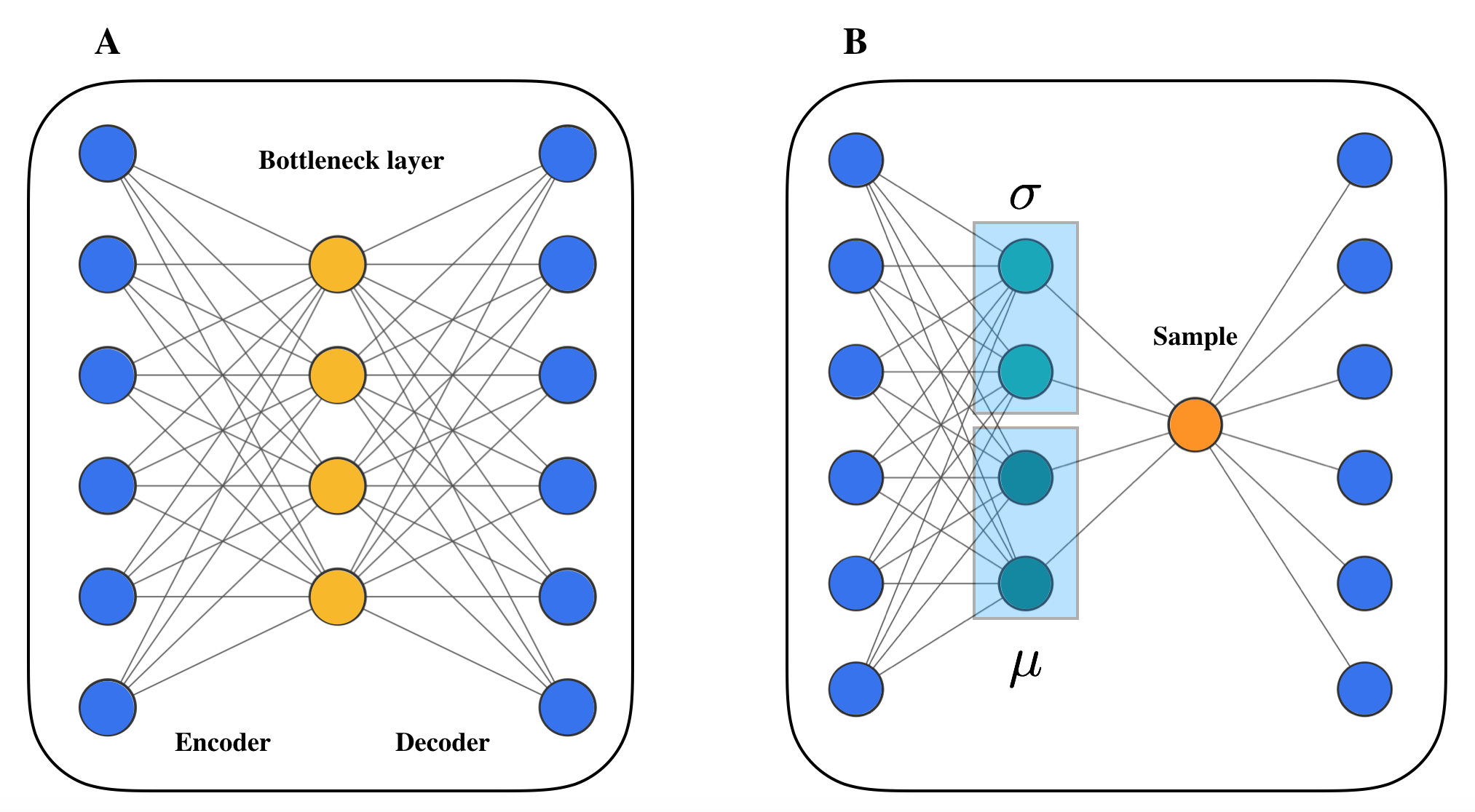

Supplement: Supplementary file 1 [file Data_Sheet_1.zip › supplementary/ae.png]

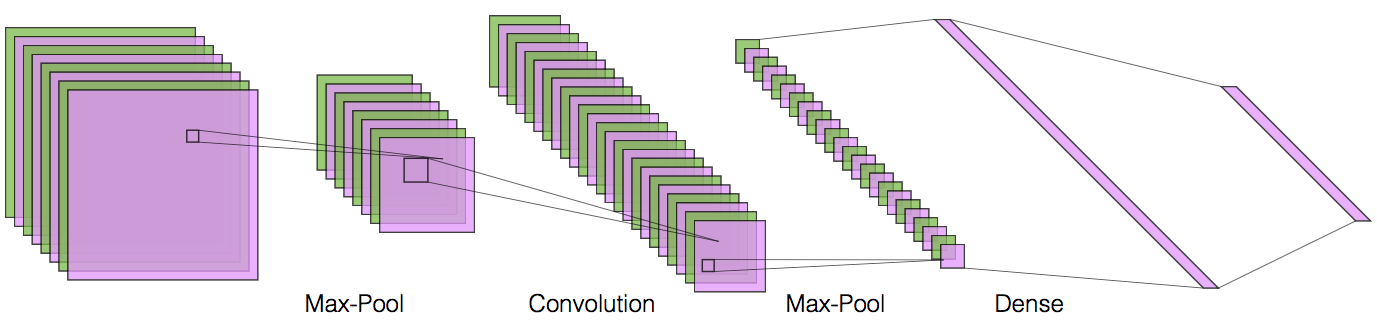

Supplement: Supplementary file 1 [file Data_Sheet_1.zip › supplementary/CNN.png]

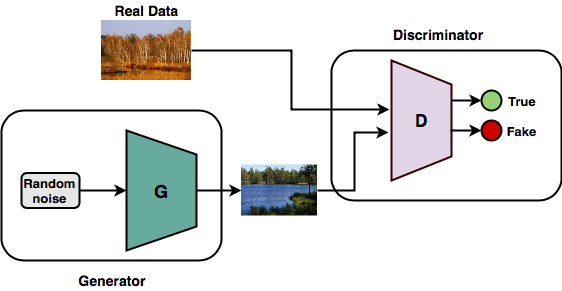

Supplement: Supplementary file 1 [file Data_Sheet_1.zip › supplementary/GAN.png]

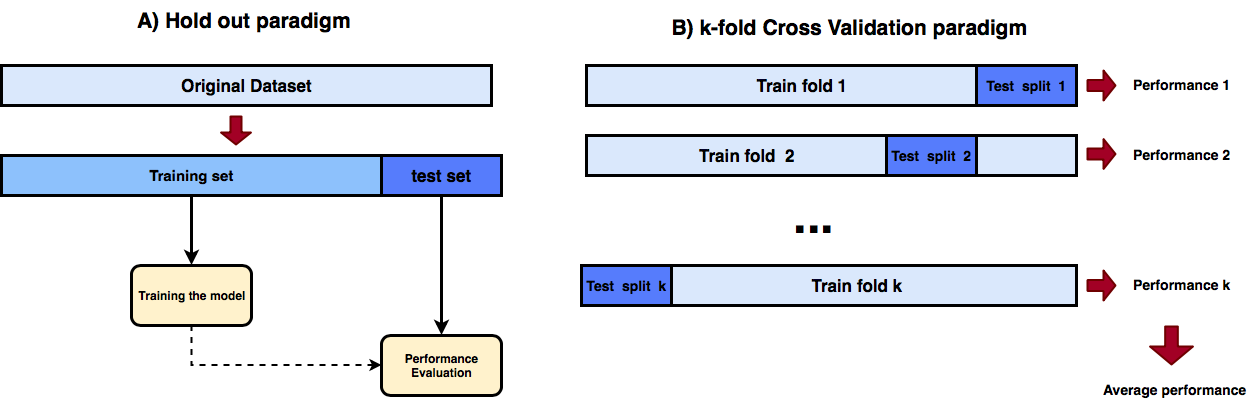

Supplement: Supplementary file 1 [file Data_Sheet_1.zip › supplementary/k-dolf-holdout.png]

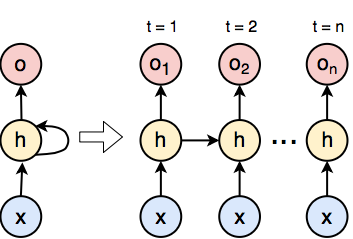

Supplement: Supplementary file 1 [file Data_Sheet_1.zip › supplementary/RNN_LSTM.png]
